# Facilitation of Behavioral and Cortical Emergence from Isoflurane Anesthesia by GABAergic Neurons in Basal Forebrain

**DOI:** 10.1523/JNEUROSCI.0628-22.2023

**Published:** 2023-04-19

**Authors:** Ping Cai, Wei-Kun Su, Jin-Sheng Zhang, Pei-Chang Liu, Feng Liu, Ren-Fu Liu, Zhang-Shu Li, Zhong-Hua Zhu, Wen-Hao Xiao, Yong-Huai Hu, Hong-Da Cai, Xiao-Dan Wu, Liang-Cheng Zhang, Changxi Yu, Li Chen

**Affiliations:** ^1^Department of Pharmacology, School of Pharmacy, Fujian Medical University, Fuzhou, Fujian 350108, China; ^2^Fujian Province Key Laboratory of Environment and Health, School of Public Health, Fujian Medical University, Fuzhou, Fujian 350108, China; ^3^Department of Anesthesiology, Fujian Medical University Union Hospital, Fuzhou, Fujian 350001, China; ^4^Department of Anesthesiology, Shengli Clinical Medical College of Fujian Medical University, Fujian Provincial Hospital, Fuzhou, Fujian 350001, China; ^5^School of Basic Medical Sciences, Fujian Medical University, Fuzhou, Fujian 350108, China; ^6^Department Of Anesthesiology, First Affiliated Hospital of Fujian Medical University, Fujian Medical University, Fuzhou, Fujian 350001, China; ^7^Fujian Key Laboratory of Drug Target Discovery and Structural and Functional Research, Fuzhou, Fujian 350108, China

**Keywords:** basal forebrain, fiber photometry, GABAergic neurons, general anesthesia, isoflurane anesthesia, optogenetics

## Abstract

General anesthesia shares many similarities with natural sleep in behavior and electroencephalogram (EEG) patterns. The latest evidence suggests that general anesthesia and sleep–wake behavior may share overlapping neural substrates. The GABAergic neurons in the basal forebrain (BF) have recently been demonstrated to play a key role in controlling wakefulness. It was hypothesized that BF GABAergic neurons may participate in the regulation of general anesthesia. Here, using *in vivo* fiber photometry, we found that the activity of BF GABAergic neurons was generally inhibited during isoflurane anesthesia, having obviously decreased during the induction of anesthesia and being gradually restored during the emergence from anesthesia, in Vgat-Cre mice of both sexes. Activation of BF GABAergic neurons with chemogenetic and optogenetic approaches decreased sensitivity to isoflurane, delayed induction, and accelerated emergence from isoflurane anesthesia. Optogenetic activation of BF GABAergic neurons decreased EEG δ power and the burst suppression ratio (BSR) during 0.8% and 1.4% isoflurane anesthesia, respectively. Similar to the effects of activating BF GABAergic cell bodies, photostimulation of BF GABAergic terminals in the thalamic reticular nucleus (TRN) also strongly promoted cortical activation and behavioral emergence from isoflurane anesthesia. Collectively, these results showed that the GABAergic BF is a key neural substrate for general anesthesia regulation that facilitates behavioral and cortical emergence from general anesthesia via the GABAergic BF-TRN pathway. Our findings may provide a new target for attenuating the depth of anesthesia and accelerating emergence from general anesthesia.

**SIGNIFICANCE STATEMENT** The basal forebrain (BF) is a key brain region controlling sleep–wake behavior. Activation of GABAergic neurons in the BF potently promotes behavioral arousal and cortical activity. Recently, many sleep–wake-related brain structures have been reported to participate in the regulation of general anesthesia. However, it is still unclear what role BF GABAergic neurons play in general anesthesia. In this study, we aim to reveal the role of BF GABAergic neurons in behavioral and cortical emergence from isoflurane anesthesia and elucidate the underlying neural pathways. Understanding the specific role of BF GABAergic neurons in isoflurane anesthesia would improve our understanding of the mechanisms of general anesthesia and may provide a new strategy for accelerating emergence from general anesthesia.

## Introduction

General anesthesia has been used in modern medicine for >170 years (Ortega et al., 2008), benefiting millions of patients who undergo surgical procedures every year; however, the precise mechanisms through which general anesthesia induces reversible unconsciousness remain unclear. There are several similarities between general anesthesia and natural sleep (Brown et al., 2010; Q. Yang et al., 2022), where the reversible loss of consciousness (LOC) is the most obvious behavioral feature shared by general anesthesia and sleep. Besides, the electroencephalogram (EEG) pattern also reflects the common characteristic. Large-amplitude slow-δ oscillations are the prominent feature shared by nonrapid eye movement (NREM) sleep and general anesthesia induced by dexmedetomidine, propofol, and isoflurane (Akeju et al., 2014, 2016a, b). Furthermore, neuroimaging evidence suggests that the cerebral functional connectivity and metabolic activity are similar during sleep and general anesthesia (Braun et al., 1997; White and Alkire, 2003; Picchioni et al., 2014; MacDonald et al., 2015). Considering the similarities between general anesthesia and sleep, recent studies have focused on the sleep–wake systems. Many brain structures related to the sleep–wake cycles have been found to participate in the regulation of general anesthesia (Bao et al., 2023), such as the basal forebrain (BF; L. Wang et al., 2021), lateral hypothalamus (LH; Herrera et al., 2016), and parabrachial nucleus (PBN; T.X. Wang et al., 2019).

The BF is a key component of the ascending arousal system and has long been implicated in sleep–wake behavior (Saper et al., 2010; Han et al., 2014; Weber and Dan, 2016). The BF mainly contains three neural subtypes: cholinergic, glutamatergic, and GABAergic neurons (Agostinelli et al., 2019). Neuroanatomical evidence has shown that the GABAergic neurons in the BF are widely interconnected with arousal-related structures (Do et al., 2016; Agostinelli et al., 2019). For example, the BF GABAergic neurons receive afference from the nucleus accumbens, central amygdala, periaqueductal gray, and parabrachial nucleus (Do et al., 2016), while they send efference to the thalamic reticular nucleus (TRN), lateral hypothalamus, ventral tegmental area, and supramammillary nucleus (Agostinelli et al., 2019). Recent studies showed that chemogenetic activation of BF GABAergic neurons produced sustained wakefulness and high-frequency cortical rhythms, whereas chemogenetic inhibition of these neurons decreased wakefulness total amount in mice (Anaclet et al., 2015). This anatomic and functional evidence illustrates that BF GABAergic neurons are critical in controlling arousal and cortical activation. However, the specific role of BF GABAergic neurons in the regulation of general anesthesia remains to be elucidated.

In the present study, using fiber photometry and synchronous electroencephalogram (EEG)/electromyogram (EMG) recording, we investigated the activity of BF GABAergic neurons during isoflurane anesthesia in Vgat-Cre mice. Then, we manipulated the activity of BF GABAergic neurons with optogenetic and chemogenetic approaches and determined its effects on behavioral responses under isoflurane anesthesia. Moreover, the effects of activating BF GABAergic neurons on cortical activity were investigated. Finally, the emergence-promoting region downstream of the GABAergic BF was identified, that is, the thalamic reticular nucleus (TRN). Collectively, our results identify the GABAergic BF as a key neural substrate controlling general anesthesia that facilitates behavioral and cortical emergence from isoflurane anesthesia via the GABAergic BF-TRN pathway.

## Materials and Methods

### Animals

Vgat-IRES-Cre mice (Strain 129S6/SvEvTac) were purchased from The Jackson Laboratory. Male and female Vgat-IRES-Cre mice aged 8–12 weeks (22–28 g) were used in this study. Approximately half of the male and half of the female mice were randomly assigned to groups of a predetermined sample size (8–10 mice in each group) to minimize the effect of gender difference on experimental results. Animals were housed under a standard environment (at an ambient temperature of 24 ± 0.5°C, relative humidity of 55 ± 5%, and 12/12 h light/dark cycle (lights on at 7 A.M.), with *ad libitum* access to water and food. All of the experimental manipulations of animals were conducted in accordance with the guidelines described in the National Institutes of Health *Guide for the Care and Use of Laboratory Animals* and were approved by the Institutional Animal Care and Use Committee of Fujian Medical University (FJMU IACUC 2021-J-0550).

### Virus and chemicals

The viruses used in the study included AAV-hSyn-DIO-GCaMP6m, AAV-Ef1α-DIO-ChR2-mCherry, AAV-hSyn-DIO-hM3Dq-mCherry, AAV-Ef1α-DIO-mCherry, and AAV-hSyn-DIO-mGFP-T2A-Synaptophysin-mRuby. All of the viruses were purchased from BrainVTA and TaiTool, and titers of all of the viruses were between 2 and 6 × 10^12^ particles/ml. Isoflurane was purchased from RWD Co, Ltd. Clozapine-N-oxide (CNO) was obtained from LKT Labs. c-Fos antibody was purchased from Abcam (catalog #Ab190289), and fluorescent goat anti-rabbit IgG was purchased from The Jackson Laboratory (catalog #111-545-003). OCT was purchased from SAKURA (catalog #4583).

### Virus injection and optical fiber implantation

Mice were anesthetized with 1.5–3% isoflurane and placed on a stereotaxic apparatus (RWD Life Science); then, 1.5% isoflurane was maintained throughout the whole surgery. The skin was cut along the center line, and small craniotomy holes were made above the BF after location according to the bregma. AAV vectors carrying different exogenous genes were slowly microinjected (50 nl/min) unilaterally or bilaterally into the BF (200 nl for each position; AP = 0.00 mm; ML = ±1.40 mm; DV = 5.5 mm). The glass pipette was left in the injection location for an additional 10 min to allow virus diffusion, then moved upward by 0.1 mm and withdrawn slowly after a 5 min stay.

After virus injection was completed, optical fibers were implanted into the target brain regions for *in vivo* fiber photometry recording and optogenetic experiments as previously described (T.X. Wang et al., 2019; Cai et al., 2020). Optical fibers (Newdoon) were placed above the BF and TRN (AP = −0.85 mm, ML = ±1.55 mm, DV = −3.40 mm) and were secured to the skull with dental cement. After the implantation of optical fibers, the EEG/EMG electrodes were implanted on the skull and fixed with dental cement. After surgery, mice were kept in a warm environment until they were fully conscious and recovered from anesthesia.

### Fiber photometry recording

First, the mice were placed in an anesthesia induction chamber (L × W × H = 23 × 10 × 17 cm) made of acrylic material and connected with the anesthesia machine. After the mice had acclimated to the environment, a baseline of the calcium signal was recorded for 30 min with the valve open for continuous delivery of 1.0% isoflurane for 30 min and continued for 30 min after the isoflurane gas delivery was turned off. We recorded the calcium signal for 90 min (30 min before anesthesia, 30 min during anesthesia, and 30 min after anesthesia each) and calculated the average ΔF/F values before, during, and after anesthesia.

We pinpointed the occurrence of LOC and recovery of consciousness (ROC) by utilizing EEG/EMG recording and further analyzed calcium signal changes during the stage of losing and recovering consciousness under 1.0% isoflurane anesthesia. The occurrence of LOC is determined by the change from low-amplitude and high-frequency EEG to high-amplitude and low-frequency EEG, as well as minimal muscle activity in the EMG (Bao et al., 2021). We continuously calculated calcium signals for three consecutive periods (from −300 to 600 s; 0 s was the time point when 1.0% isoflurane was on): −300–0 s (baseline before anesthesia), pre-LOC (from 0 s to LOC), and post-LOC (from LOC to 600 s). At the same time, the occurrence of ROC was determined by the transformation of the EEG signal to a low amplitude and high frequency and the enhancement of muscle activity signaling in EMG. We continuously calculated calcium signals for three consecutive periods (from −300 to 600 s; 0 s was the time point when 1.0% isoflurane was off): unconsciousness anesthesia baseline (−300–0 s), pre-ROC (from 0 s to ROC), and post-ROC (from ROC to 600 s). In addition, we calculated the baseline and average calcium signal in each state. Details of the fiber photometry recording method are provided in the Extended Data 1.

10.1523/JNEUROSCI.0628-22.2023.ed1 Extended Data 1 Supplemental materials and methods. Details of the methods of fiber photometry recording, estimation of induction and emergence, arousal scoring during photostimulation, burst suppression ratio (BSR) analysis, and immunofluorescence. Download Extended Data 1, DOCX file.

### Estimation of induction and emergence from isoflurane anesthesia

The righting reflex test was used to evaluate the anesthetic effects of isoflurane based on previous studies (Yin et al., 2019; Bao et al., 2021). In chemogenetic experiments, 1 mg/kg CNO or vehicle (the same volume) was intraperitoneally injected into the mice 1 h before the experiment. The mice were placed in an anesthesia induction chamber filled with 1.4% isoflurane at a flow rate of 1.5 l/min. The concentration of isoflurane in the chamber was monitored with an infrared gas analyzer (G60; Philips) in the outflow of the chamber. The chamber was gently rotated 90° every 10 s and then slowly returned to the original position; the time when the mice were unable to turn themselves to a prone position within 30 s was regarded as the occurrence of the loss of righting reflex (LORR; T.X. Wang et al., 2019; Yin et al., 2019). The time from the onset of isoflurane exposure to the occurrence of LORR was recorded as the induction time. After 30 min of isoflurane exposure, the isoflurane was shut off. Mice were removed from the chamber and rapidly exposed to air until they experienced a recovery of the righting reflex (RORR; T.X. Wang et al., 2019; Yin et al., 2019). The time from the cessation of isoflurane exposure to the occurrence of RORR was recorded as the emergence time. In all of the experiments, the body temperature of the mice was maintained by placing a 37°C heating pad under the chamber.

To determine the dose–response curve for LORR, isoflurane was initially delivered at a concentration of 0.5% and was increased in 0.1% increments every 15 min until mice developed LORR. In experiments to determine dose–response curves for RORR, isoflurane was initially delivered at a concentration of 1.4% and reduced in 0.1% increments, tapering every 15 min until mice developed RORR. All of the behavioral experiments were conducted by an experienced researcher blinded to mouse groups and performed between 9 A.M. and 5 P.M., with at least a 3-d experimental interval. The details of the estimation of induction and emergence in the optogenetic and chemogenetic experiments are provided in the Extended Data 1.

### Arousal scoring during photostimulation

We also evaluated the effect of a sequence of light pulses (10-ms pulses at 5–20Hz for 60 s) on behavioral responses in mice under lighter isoflurane anesthesia. Briefly, each mouse was placed supine in the center of the chamber and exposed to 1.4% isoflurane for 30 min. The isoflurane concentration was then reduced to 0.6%. If the mouse showed any signs of RORR, the chamber concentration increased by 0.1% until the mouse maintained LORR for 30 min continuously. Therefore, the isoflurane concentration, ranging from 0.6% to 0.7%, slightly varied depending on the behavior of each mouse, as previously described (Bao et al., 2021). Following a 30 min equilibration period, acute photostimulation (10-ms pulses at 5–20 Hz for 60 s) was initiated, while mice continued to inhale the same concentration of isoflurane during the photostimulation, and arousal responses were then scored. The procedural details for arousal scoring are provided in the Extended Data 1.

### Optogenetic manipulations during 0.8% and 1.4% isoflurane anesthesia

The electrodes and optical fibers of mice were, respectively, connected with the amplifier and light source. The mice were placed in the anesthesia induction chamber. After the habituation of mice in the chamber, 0.8% or 1.4% of isoflurane gas was delivered at 1.5 l/min for induction. Photostimulation was given for 120 s (10 ms; 20 Hz; 3–5 mW) after the isoflurane concentration in the chamber was stabilized at 0.8% or 1.4% for 30 min. In the experiment of 1.4% isoflurane anesthesia, a stable burst suppression oscillation mode was recorded for at least 5 min before photostimulation. The administration of isoflurane was ceased 10 min after the laser was turned off, and the mouse was then removed from the chamber. EEG and EMG were recorded throughout the experiment.

### EEG/EMG recordings and analysis

All of the EEG/EMG signals during optogenetic experiments were recorded and analyzed by the SleepSign software (Kissei Comtec). The amplified EEG/EMG data were collected at 128 Hz sampling rate. In the experiment exploring the effect of optogenetics on cortical EEG under 0.8% isoflurane anesthesia, the EEG frequency band (δ, 0.5–4.0 Hz; θ, 4.0–7.0 Hz; α, 8.0–15.0 Hz; β, 16.0–30.0 Hz) used fast Fourier transform to calculate the relative change in the total power of BF-GABAergic neurons within 120 s before, during, and after photostimulation. The burst suppression ratio (BSR) is a widely adopted method for quantifying burst suppression. For the optogenetic experiments of burst suppression oscillation, raw EEG data recorded by SleepSign software were converted to text format for further analysis of BSR using MATLAB R2019b (Bao et al., 2021). Finally, the BSR changes in BF-GABAergic neurons within 120 s before, during, and after photostimulation were calculated. The details of BSR analysis are provided in the Extended Data 1.

### Data analysis and statistics

All of the data are shown as mean ± SEM. The two-tailed unpaired *t* test, paired *t* test, two-tailed Wilcoxon rank-sum test, and one-way or two-way repeated measures (RM) ANOVA with a following Bonferroni's multiple comparisons test were used to assess statistical significance based on GraphPad 8.0. Significance was defined as *p* < 0.05. Significance annotations as follows: *p < 0.05, **p < 0.01, ***p < 0.001. The layout of all of the figures was generated by Adobe Illustrator.

## Results

### The activity of BF GABAergic neurons decreased during isoflurane anesthesia

**Figure 1.**
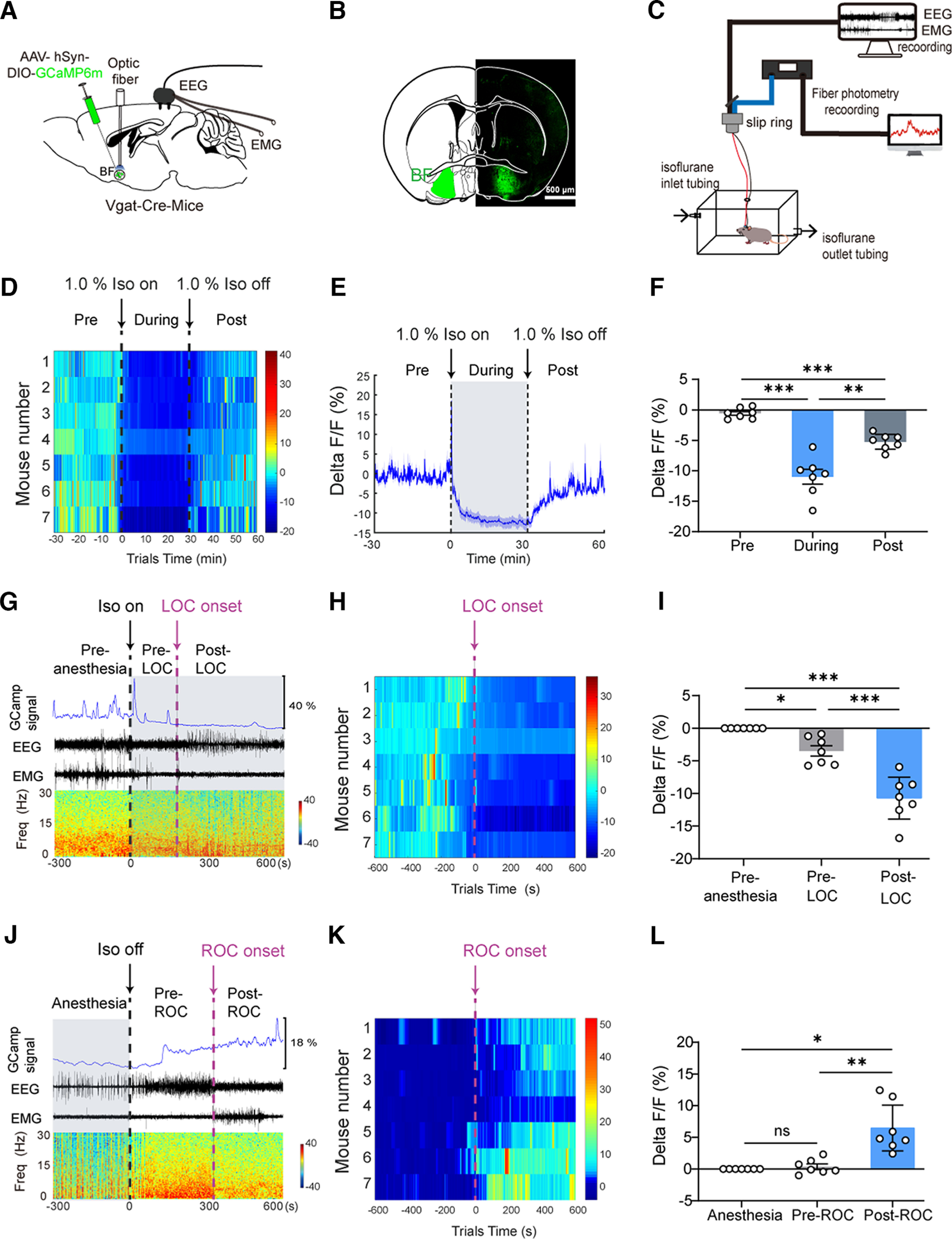
The activity of BF GABAergic neurons is inhibited during isoflurane anesthesia. ***A***, Schematic diagram of AAV-hSyn-DIO-GCaMP6m (green) injected into the BF of Vgat-Cre mice. ***B***, Representative coronal image showing the expression GCaMP6m in the BF GABAergic neurons (scale bar, 500 μm). ***C***, Schematic of fiber photometry recording in isoflurane anesthesia. ***D***, Heatmaps for the calcium signals of BF GABAergic neurons during general anesthesia induced by 1.0% isoflurane. 0 represents the moment of 1.0% isoflurane on. ***E***, Time courses of calcium signals following 1.0% isoflurane anesthesia (*n* = 7). ***F***, Quantification of calcium signals changes before, during, and after isoflurane inhalation (*n* = 7). ***G***, Schematic diagram shows the calcium signal trace aligned with the LOC state transition. 0 represents the moment of 1.0% isoflurane on. ***H***, Heatmaps for the calcium signals of BF GABAergic neurons during LOC induced by 1.0% isoflurane. 0 represents the moment of LOC onset. ***I***, Quantification of calcium signal changes in three consecutive time periods of LOC (*n* = 7). ***J***, Schematic diagram shows the calcium signal trace aligned to ROC state transition. ***K***, Heatmaps for the calcium signals of BF GABAergic neurons during ROC after isoflurane exposure. 0 represents the moment of ROC onset. ***L***, Quantification of calcium signal changes in three consecutive time periods of ROC (*n* = 7). The asterisk in ***F***, ***I***, and ***L***, indicates a significant difference (*p < 0.05, **p < 0.01, ***p < 0.001). Statistical comparisons were conducted using one-way ANOVA in ***F***, ***I***, and ***L*** followed by Bonferroni's *post hoc* test. Error bars represent ± SEM. EEG, electroencephalogram; EMG, electromyogram; b.s., baseline; Freq, frequency; LOC, loss of consciousness; ROC, recovery of consciousness; Iso, isoflurane. Additional information to support this figure can be found in the Extended Data Figure 1-1.

To investigate whether the activity of BF GABAergic neurons is correlated with isoflurane anesthesia, we used fiber photometry to detect the calcium signals of these neurons in freely behaving mice (Fig. 1*C*). The AAV-hSyn-DIO-GCaMP6m was injected into the BF of Vgat-Cre mice, and an optical fiber was implanted above the viral injection site (Fig. 1*A*). The electrodes were implanted in the cerebral cortex and posterior cervical muscle of mice to record EEG/EMG simultaneously (Fig. 1*A*). Four weeks after virus transfection, robust expression of GCaMP6m was observed in the BF (Fig. 1*B*; Extended Data Fig. 1-1). We examined BF GABAergic neuronal activity during the exposure of isoflurane. The results of fiber photometry showed that the GCaMP6m fluorescence dynamically fluctuated across the isoflurane exposure (Fig. 1*D*,*E*). When the mice were exposed to 1.0% isoflurane, the GCaMP6m signals gradually decreased after a transient peak period (Fig. 1*E*). After 15 min of isoflurane exposure, the GCaMP6m signals were stable at −11% (Fig. 1*E*). After the cessation of isoflurane administration, the GCaMP6m signals were slowly restored to the preanesthesia level (Fig. 1*E*). Statistical results showed that the activity of BF GABAergic neurons significantly decreased during the 30 min exposure to isoflurane (*F*_(1.299,7.795)_ = 47.44, *p* < 0.0001, one-way ANOVA followed by Bonferroni's *post hoc* test; pre–during comparison, from −0.57 ± 0.33% to −10.96 ± 1.23%, *p* = 0.0007; Fig. 1*F*) and significantly increased after the cessation of isoflurane exposure (during–post comparison, from −10.96 ± 1.23% to −5.23 ± 0.49%, *p* = 0.0075; Fig. 1*F*).

10.1523/JNEUROSCI.0628-22.2023.f1-1 Extended Data Figure 1-1 Drawings of superimposed expression of AAV-hSyn-DIO-GCaMP6m in the BF. AAV-hSyn-DIO-GCaMP6m was injected into the BF of Vgat-Cre mice, and expression of GCaMP6m was checked after behavioral testing (*n* = 7, indicated with different colors). Download Figure 1-1, TIF file.

Next, we further analyzed the change in neuronal activity during the induction and emergence from isoflurane anesthesia by synchronously recording EEG/EMG signals with a polygraphic recording system (Fig. 1*C*). For the induction period, we calculated the calcium signals in three consecutive time periods: the preanesthesia period (−300–0 s; 0 s represents the time when 1.0% isoflurane was given), the period before loss of consciousness (pre-LOC, from 0 to LOC), and the period after LOC (post-LOC, from LOC to 600 s). Our results showed that, after the mice were exposed to isoflurane, the amplitude of EEG and activity of EMG obviously decreased (Fig. 1*G*). The loss of consciousness (LOC) was identified ∼200 s after isoflurane exposure (Fig. 1*G*), which corresponded to a transition of EEG pattern into a high-amplitude and low-frequency oscillation, with a continuously minimal muscle tone indicated in the EMG (Jiang-Xie et al., 2019). The heatmap showed that GCaMP6m signals obviously decreased after the onset of LOC (Fig. 1*H*). Statistical results showed that BF GABAergic neuronal activity decreased by ∼3% in the pre-LOC period (*F*_(1.465,8.788)_ = 55.06, *p* < 0.0001, one-way ANOVA followed by Bonferroni's *post hoc* test; pre-LOC vs preanesthesia, −3.49 ± 0.79%, *p* = 0.0138; Fig. 1*I*) and further decreased by ∼7% in the post-LOC period (post-LOC vs pre-LOC, 7.22 ± 0.95%, *p* = 0.0008; Fig. 1*I*).

For the emergence period, we analyzed three consecutive periods of the calcium signals in three consecutive time periods: the unconsciousness period (−300 s to 0 s; 0 s represents the time when 1.0% isoflurane was turned off), the period before recovery of consciousness (pre-ROC, from 0 s to ROC), and the period after ROC (post-ROC, from ROC to 600 s). Our results showed that, ∼300 s after the cessation of isoflurane administration, the EEG converted into a low-amplitude, high-frequency oscillation, and the activity of EMG obviously increased (Fig. 1*J*), indicating the recovery of consciousness in mice (Jiang-Xie et al., 2019). The heatmap showed that GCaMP6m signals obviously increased after the onset of ROC (Fig. 1*K*). Statistical results showed that BF GABAergic neuronal activity significantly increased by ∼6% in the period after ROC (*F*_(1.120,6.721)_ = 19.60, *p* = 0.0030, one-way ANOVA followed by Bonferroni's *post hoc* test; post-ROC vs pre-ROC, 6.09 ± 1.29%, *p* = 0.0098; Fig. 1*L*). Taken together, these results indicate that BF GABAergic neuron activity is closely related to isoflurane anesthesia. The significant change in neuronal activity during the LOC and ROC suggests that BF GABAergic neurons may play an important role in the regulation of isoflurane anesthesia.

### Chemogenetic activation of BF GABAergic neurons delayed induction and accelerated emergence from isoflurane anesthesia

**Figure 2.**
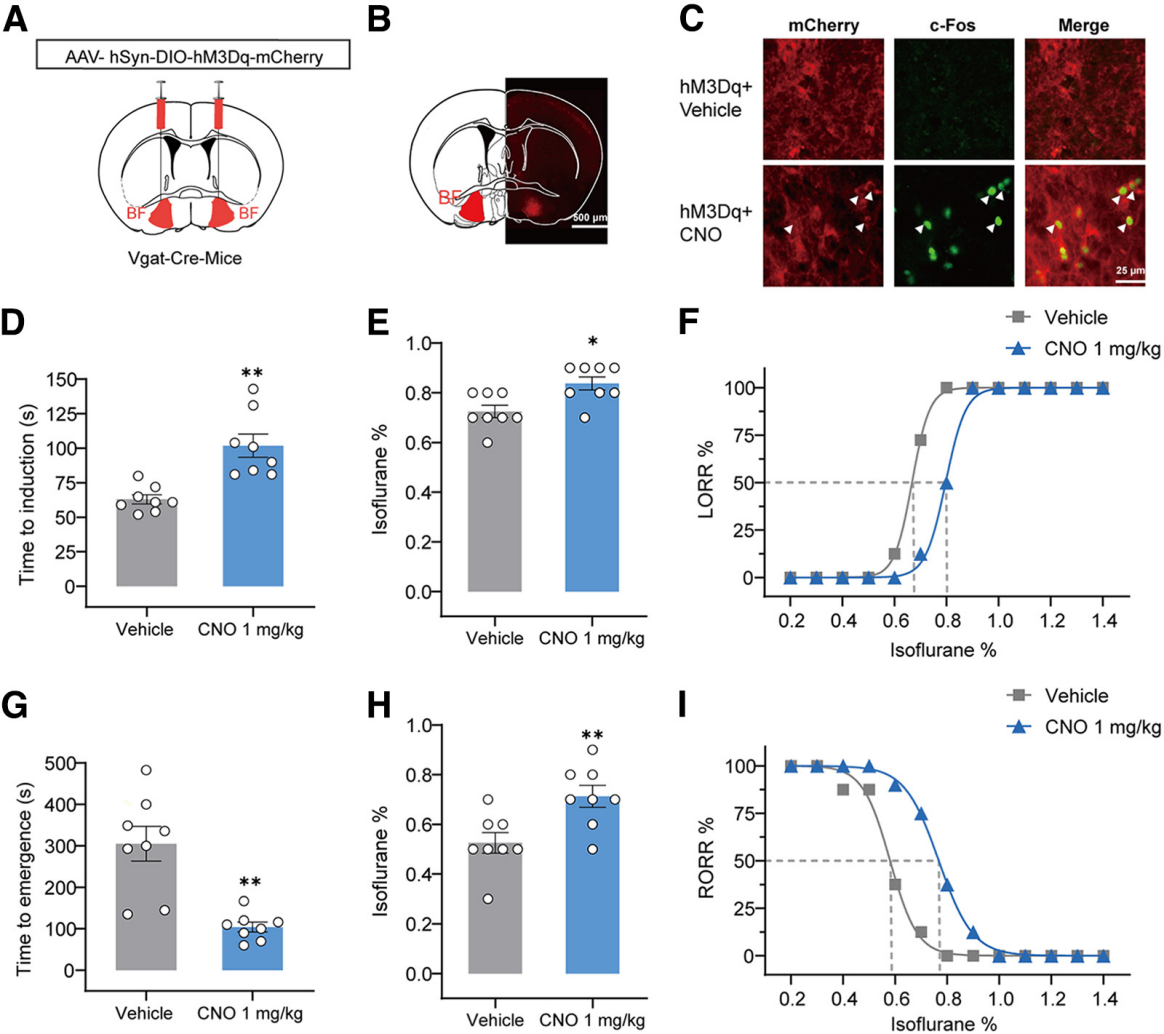
Chemogenetic activation of BF GABAergic neurons delays the induction and facilitates the emergence from isoflurane anesthesia. ***A***, Schematic diagram of injection of AAV-hSyn-DIO-hM3Dq-mCherry into the BF of Vgat-Cre mice. ***B***, Representative image showing the expression hM3Dq in the BF GABAergic neurons (scale bar, 500 μm). ***C***, Representative images of mCherry/c-Fos immunofluorescence in the BF after vehicle or CNO treatment. White arrowheads highlight merged signals (scale bar: 25 μm). ***D***, Effects of chemogenetic activation of BF GABAergic on the time of LORR under 1.4% isoflurane anesthesia (*n* = 8). ***E***, Chemogenetic activation of BF GABAergic neurons increased the isoflurane concentration inducing LORR (*n* = 8). ***F***, Dose-response curve shows the percentage of mice showing LORR in response to incremental isoflurane concentration after vehicle and CNO injection (*n* = 8). ***G***, Effects of chemogenetic activation of BF GABAergic on the time of RORR under 1.4% isoflurane anesthesia. ***H***, Chemogenetic activation of BF GABAergic neurons increased the isoflurane concentration at which mice showed RORR (*n* = 8). ***I***, Dose-response curve shows the percentage of mice showing RORR with gradually decreased isoflurane concentration after vehicle and CNO injection (*n* = 8). The asterisk in ***D–I*** indicates a significant difference (*p < 0.05, **p < 0.01). Statistical comparisons were conducted using Student's two-tailed paired *t* test (***D***, ***G***) or Wilcoxon signed-rank test (***E***, ***H***). Error bars represent ± SEM. LORR, loss of righting reflex; RORR, recovery of righting reflex; CNO, clozapine-N-oxide. Additional information to support thus figure can be found in the Extended Data Figures 2-1 and 2-2.

Next, we determined whether BF GABAergic neurons participate in the regulation of isoflurane anesthesia. We examined the effect of chemogenetic activation of BF GABAergic neurons. AAV-DIO-hM3Dq-mCherry was injected into the BF of Vgat-Cre mice (Fig. 2*A*). About four weeks later, a strong expression of hM3Dq-mCherry was observed in the BF (Fig. 2*B*; Extended Data Fig. 2-1). Immunofluorescent staining results showed that more c-Fos protein was expressed in hM3Dq-mCherry-positive neurons following 1 mg/kg CNO injection compared with vehicle administration (Fig. 2*C*), whereas sparse c-Fos protein was expressed following vehicle administration. The function of hM3Dq was confirmed via an *in vitro* electrophysiological experiment (Extended Data Fig. 2-2*A*). Whole-cell current-clamp recordings showed that the application of 5 μm CNO potently increased the firing frequency of BF GABAergic neurons (Extended Data Fig. 2-2*B*), indicating the activation of BF GABAergic neurons by chemogenetics. We first conducted the righting reflex test to determine the effect of GABAergic BF activation on isoflurane anesthesia. LORR and RORR in rodents are regarded as a surrogate for anesthesia induction and emergence in humans, respectively (Pal et al., 2015; Herrera et al., 2016). Our results showed that, when the mice were exposed to 1.4% isoflurane, 1 mg/kg CNO injection significantly prolonged the induction time from 63 ± 3 to 102 ± 8 s (*t*_(7)_ = 4.05, *p* = 0.0049; Fig. 2*D*) and shortened the emergence time from 305 ± 42 to 104 ± 12 s (*t*_(7)_ = 5.19, *p* = 0.0013; Fig. 2*G*) compared with vehicle injection. We further determined the effect of BF GABAergic activation on isoflurane sensitivity by progressively changing the isoflurane concentration. Chemogenetic activation of BF GABAergic neurons rendered a higher isoflurane concentration needed to induce LORR in mice, which increased from 0.725 ± 0.025% to 0.838 ± 0.026% (*W* = 28, *p* = 0.0156; Fig. 2*E*). Moreover, CNO injection led to a rightward shift of the dose–response curve for LORR (Fig. 2*F*). The minimum concentration of isoflurane contributing to the LORR of half the mice, that is, the 50% effective concentration (EC_50_) of isoflurane on LORR, significantly increased to 0.797% (95% confidence index 0.786–0.804%) after CNO injection, compared with 0.667% (95% confidence index 0.664–0.669%) after vehicle injection (Fig. 2*F*).

10.1523/JNEUROSCI.0628-22.2023.f2-1 Extended Data Figure 2-1 Drawings of superimposed expression of AAV-hSyn-DIO-hM3Dq-mCherry in the BF. AAV-hSyn-DIO-hM3Dq-mCherry was injected into the BF of Vgat-Cre mice, and the expression of hM3Dq was checked after behavioral testing (*n* = 7, indicated with different colors). Download Figure 2-1, TIF file.

10.1523/JNEUROSCI.0628-22.2023.f2-2 Extended Data Figure 2-2 *In vitro* electrophysiological results confirm the activation of BF GABAergic neurons by chemogenetic approaches. ***A***, Schematic diagram of injection of AAV-hSyn-DIO-hM3DGq-mCherry into the BF of Vgat-Cre mice to prepare brain slices *in vitro* electrophysiological experiments. ***B***, Representative voltage tracer shows that bath application of CNO increases firing rate in hM3Dq-expressing BF GABAergic neurons. Download Figure 2-2, TIF file.

In addition, chemogenetic activation of BF GABAergic neurons required a higher concentration of isoflurane to cause RORR in mice, which increased from 0.525 ± 0.041% to 0.713 ± 0.044% (*W* = 36, *p* = 0.0078; Fig. 2*H*). CNO injection caused a rightward shift of the dose–response curve for RORR and increased the EC_50_ of RORR to 0.768% (95% confidence index 0.761%–0.775%), compared with 0.580% (95% confidence index 0.565–0.601%) after vehicle injection (Fig. 2*I*) during isoflurane anesthesia. Collectively, these results indicated that chemogenetic activation of the BF GABAergic neurons altered behavioral consequences during isoflurane anesthesia, with reduced sensitivity, delayed induction, and accelerated emergency from isoflurane anesthesia.

### Optogenetic activation of BF GABAergic neurons facilitated behavioral emergence from isoflurane anesthesia

**Figure 3.**
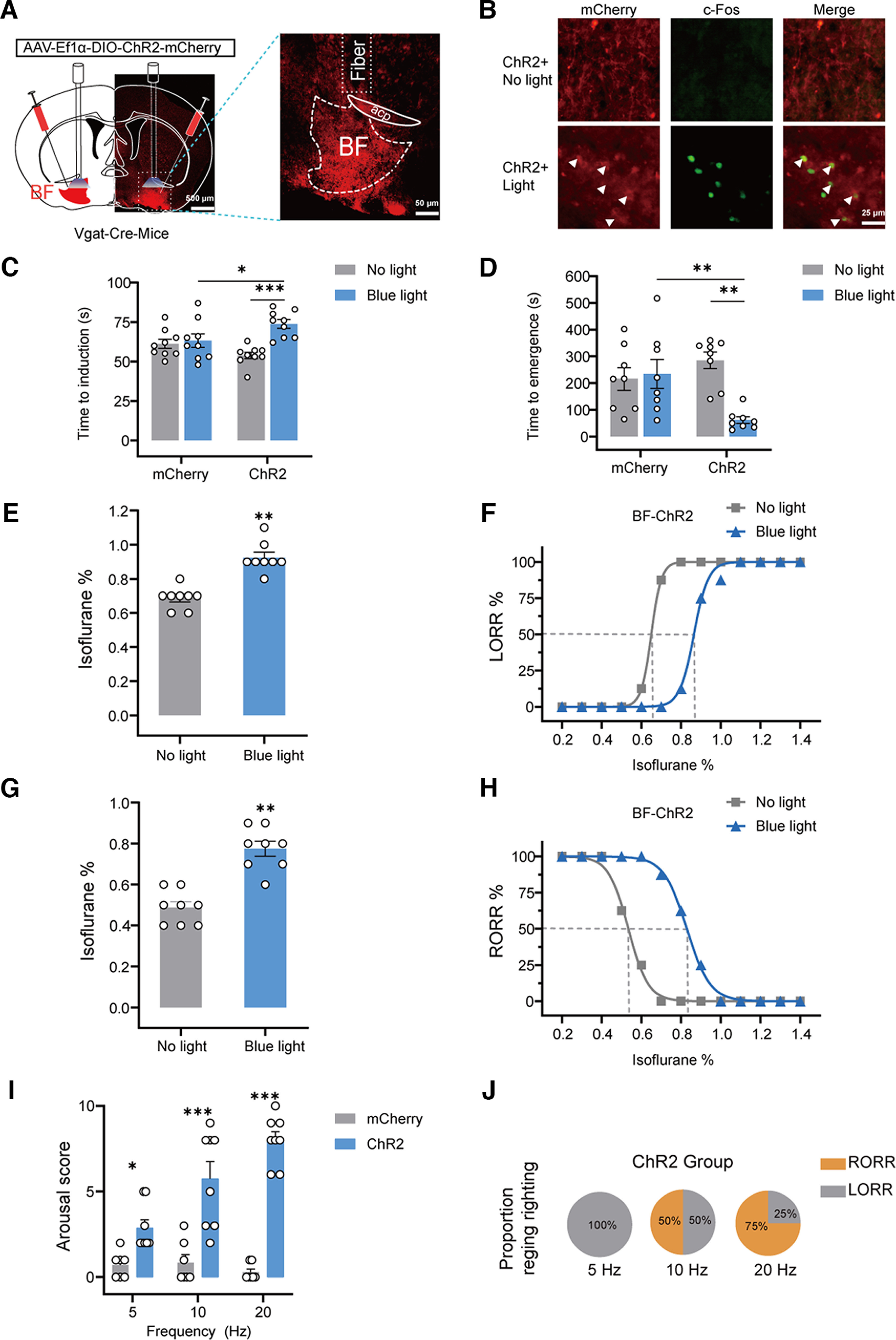
Optogenetic activation of BF GABAergic neurons delays the induction and facilitates the emergence from isoflurane anesthesia. ***A***, Representative image showing the expression of AAV-Ef1α-DIO-ChR2-mCherry and fiber position (left), and the amplified image (right). ***B***, Representative images of mCherry/c-Fos immunofluorescence in the BF after no light or blue light treatment. White arrowheads highlight merged signals (scale bar: 25 μm). ***C***, ***D***, The effect of optogenetic activation of BF GABAergic neurons on LORR (*n* = 9) and RORR (*n* = 8) time under 1.4% isoflurane anesthesia. ***E***, Photostimulation of BF GABAergic neurons increased the isoflurane concentration inducing LORR in ChR2 mice (*n* = 8). ***F***, The dose-response curve shows the percentage of mice showing LORR in response to incremental isoflurane concentration in ChR2 group with or without blue light stimulation (*n* = 8). ***G***, Photostimulation of BF GABAergic neurons increased the isoflurane concentration at which ChR2 mice showed RORR (*n* = 8). ***H***, The dose-response curve shows the percentage of mice showing RORR with gradually decreased isoflurane concentration in ChR2 group with or without blue light stimulation (*n* = 8). ***I***, Arousal scores based on the mice behavioral response to BF GABAergic neurons photostimulation (5–20 Hz, 5 ms, 60 s) under isoflurane anesthesia (*n* = 8 ChR2 mice or *n* = 7 mCherry mice per group). ***J***, Pie chart shows the proportion of ChR2 mice recovering righting reflex after photostimulation of BF GABAergic neurons at 5–20 Hz for 60 s (*n* = 8 ChR2 mice per group). The asterisk in ***C–I*** indicates a significant difference (*p < 0.05, **p < 0.01, ***p < 0.001). Statistical comparisons were conducted using Wilcoxon signed-rank test (***E***, ***G***) or two-way repeated measures ANOVA followed by Bonferroni's *post hoc* test (***C***, ***D***, ***I***). Error bars represent mean ± SEM. LORR, loss of righting reflex; RORR, recovery of righting reflex. Additional information to support thus figure can be found in the Extended Data Figures 3-1, 3-2, and 3-3.

In order to further illustrate the role of GABAergic BF in the regulation of behavioral responses during isoflurane anesthesia, we examined the effect of activation of BF GABAergic neurons with optogenetics, which has a higher temporal resolution than chemogenetics. The AAV-EF1α-DIO-ChR2-mCherry was injected into the BF of Vgat-Cre mice (ChR2 mice), and a strong expression of ChR2-mCherry was observed in the BF (Fig. 3*A*; Extended Data Fig. 3-1). Immunofluorescent staining results showed that photostimulation drove a large amount of c-Fos expression in ChR2-positive neurons (Fig. 3*B*). Functional expression of ChR2 was confirmed via an *in vitro* electrophysiological experiment (Extended Data Fig. 3-2*A*). Whole-cell voltage-clamp recordings showed that blue light stimulation induced a depolarizing current in BF GABAergic neurons (Extended Data Fig. 3-2*B*). Whole-cell current-clamp recordings showed that blue light stimulation at different frequencies evoked a series of action potentials in BF GABAergic neurons (Extended Data Fig. 3-2*C–F*), indicating the reliable activation of BF GABAergic neurons by optogenetics. Firstly we observed the effect of optogenetic activation of BF GABAergic neurons on the induction and emergence under 1.4% concentration of isoflurane anesthesia. Our results showed that light stimulation significantly increased induction time from 54 ± 2 s to 74 ± 3 s (*F*_(1,16)_ = 14.73, *p* = 0.0015, two-way repeated measures ANOVA followed by Bonferroni's *post hoc* test; ChR2-mice, *p* < 0.0001; Fig. 3*C*) and decreased the emergence time from 285 ± 31 to 61 ± 13 s in ChR2 mice (*F*_(1,14)_ = 8.58, *p* = 0.0110, two-way repeated measures ANOVA followed by Bonferroni's *post hoc* test; ChR2-mice, *p* = 0.0038; Fig. 3*D*). Photostimulation did not significantly change the induction time or emergence time in mCherry mice (Fig. 3*C*,*D*).

10.1523/JNEUROSCI.0628-22.2023.f3-1 Extended Data Figure 3-1 Drawings of superimposed expression of AAV-Ef1α-DIO-ChR2-mCherry in the BF. AAV-Ef1α-DIO-ChR2-mCherry was injected into the BF of Vgat-Cre mice, and the expression of ChR2 was checked after behavioral testing (*n* = 8, indicated with different colors). Download Figure 3-1, TIF file.

10.1523/JNEUROSCI.0628-22.2023.f3-2 Extended Data Figure 3-2 *In vitro* electrophysiological results confirm the activation of BF GABAergic neurons by optogenetic approaches. ***A***, Schematic diagram of injection of AAV-EF1α-DIO-ChR2-mCherry into the BF of Vgat-Cre mice to prepare brain slices *in vitro* electrophysiological experiments. ***B***, Representative current tracer shows that 473-nm blue light stimulation induces a depolarization current in ChR2-expressing BF GABAergic neurons. ***C–F***, Representative current-clamp recording results show that neuronal firing of ChR2-expressing BF GABAergic neurons was reliably evoked by optogenetic stimulation at different frequencies. Download Figure 3-2, TIF file.

10.1523/JNEUROSCI.0628-22.2023.f3-3 Extended Data Figure 3-3 Behavioral responses of Vgat-Cre mice during acute optogenetic activation of BF GABA neurons at 20 Hz under isoflurane anesthesia. Behavioral responses were undertaken during 60 s of acute photostimulation. Spontaneous movements of the head, tail and limbs, as well as righting reflex and walking status were scored for each mouse. The total score for each mouse depends on the sum of all categories. Download Figure 3-3, DOCX file.

Then, we detected the effect of optogenetic activation of GABAergic BF on isoflurane sensitivity. Optogenetic activation of BF GABAergic neurons rendered a higher isoflurane concentration needed to induce LORR, which increased from 0.688 ± 0.0227% to 0.925 ± 0.0313% (*W* = 36, *p* = 0.0078; Fig. 3*E*). The dose–response curve for LORR was right-shifted after photostimulation (Fig. 3*F*). The EC_50_ of LORR significantly increased from 0.650% (95% confidence interval, 0.650–0.651%) to 0.865% (95% confidence interval, 0.848–0.876%; Fig. 3*F*). Furthermore, optogenetic activation of BF GABAergic neurons led to a higher isoflurane concentration inducing RORR, which increased from 0.488 ± 0.030% to 0.775 ± 0.037% (*W* = 36; *p* = 0.0078; Fig. 3*G*). The EC_50_ of RORR increased from 0.534% (95% confidence interval 0.523–0.546%) to 0.829% (95% confidence interval 0.821–0.838%; Fig. 3*H*).

Additionally, we investigated the effect of GABAergic BF activation on the initiation of behavioral emergence according to previous studies (Taylor et al., 2016; Bao et al., 2021) After the mice were anesthetized and maintained LORR for 30 min, we applied brief photostimulation (lasting 60 s) with different frequencies (5–20 Hz) and determined the arousal scores based on the behavioral responses of mice (Bao et al., 2021). Our results showed that photostimulation significantly increased the arousal scores frequency dependently (Fig. 3*I*). Photostimulation at 20 Hz induced obvious behavioral responses in mice, including body movement (limbs, heads, and tails; 8/8), a restored righting reflex (6/8), and walking (3/8), in ChR2 mice (Extended Data Fig. 3-3). Photostimulation increased the proportion of mice regaining the righting reflex in a frequency-dependent way (Fig. 3*J*). Photostimulation at 20 Hz induced 75% mice to regain the righting reflex (Fig. 3*J*). In mCherry mice, photostimulation did not induce behavioral responses of mice under isoflurane anesthesia (Fig. 3*I*). These optogenetic results, together with the chemogenetic results above, clearly illustrated that activation of the GABAergic BF facilitates behavioral emergence from isoflurane anesthesia.

### Optogenetic activation of BF GABAergic neurons promoted cortical activation during isoflurane anesthesia

**Figure 4.**
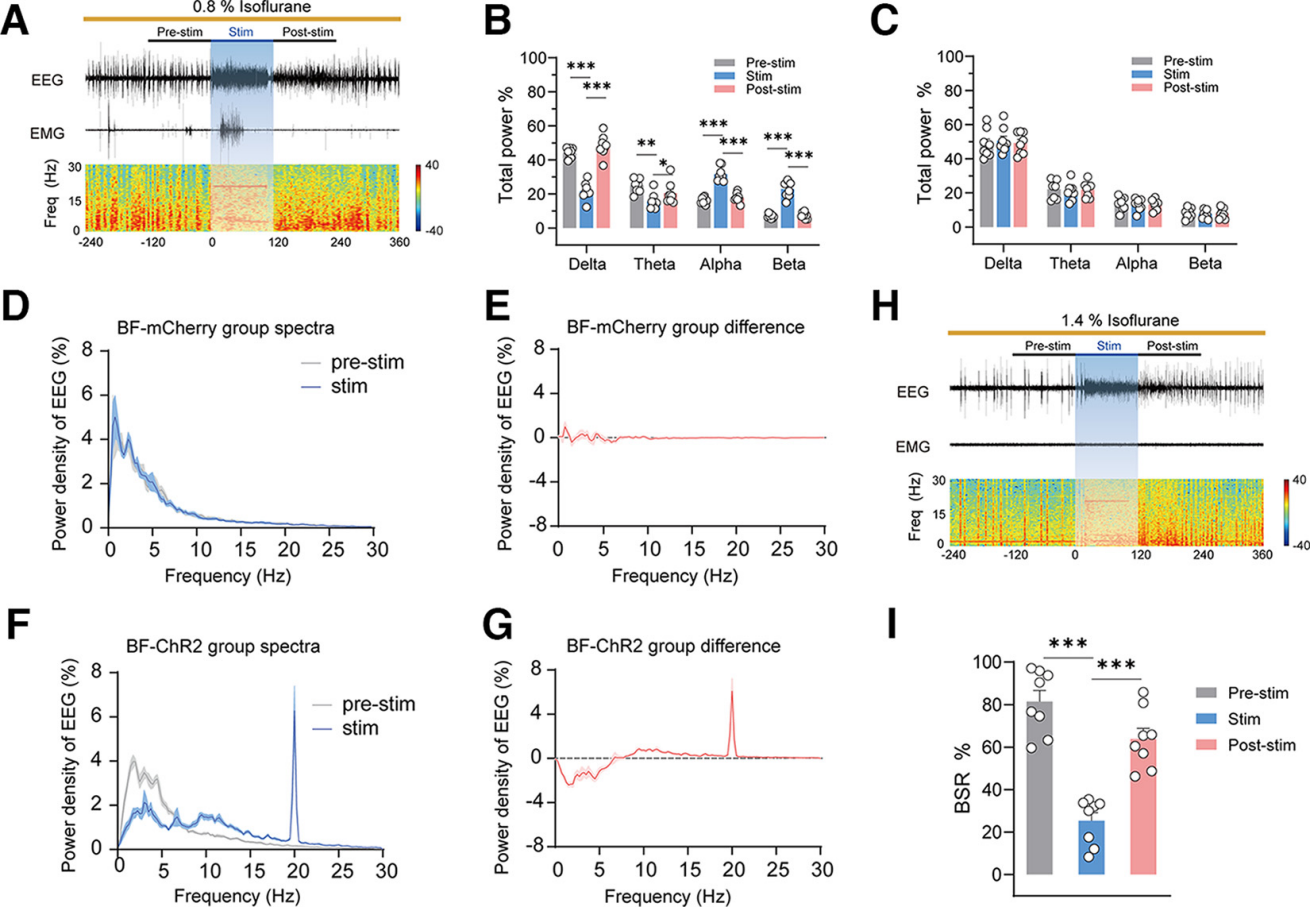
Optogenetic stimulation of BF GABAergic neurons induces rapid cortical activation during 0.8% and 1.4% isoflurane anesthesia. ***A***, Representative images showing EEG, EMG, and EEG spectrograms of a ChR2 mouse receiving acute photostimulation (20 Hz, 10 ms, 120 s) during 0.8% isoflurane anesthesia. ***B***, Relative EEG power before (gray), during (blue), and after (pink) 20-Hz photostimulation in ChR2 mice during 0.8% isoflurane anesthesia (*n* = 8). ***C***, Relative EEG power before (gray), during (blue), and after (pink) photostimulation at 20 Hz in BF-mCherry mice during isoflurane anesthesia (*n* = 8). ***D***, Normalized power densities of EEG signals before and during acute photostimulation at 20 Hz in BF-mCherry mice (*n* = 8). ***E***, Differences of normalized power densities of EEG signals before and during acute photostimulation at 20 Hz in BF-mCherry mice. ***F***, Normalized power densities of EEG signals before and during acute photostimulation at 20 Hz in BF-ChR2 mice (*n* = 8). ***G***, Differences of normalized power densities of EEG signals before and during acute photostimulation in BF-ChR2 mice. ***H***, Representative images showing EEG, EMG, and EEG spectrograms of a ChR2 mouse receiving acute photostimulation (20 Hz, 10 ms, 120 s) during 1.4% isoflurane anesthesia. ***I***, Relative BSR before (gray), during (blue), and after (pink) 20-Hz photostimulation in ChR2 mice during 1.4% isoflurane anesthesia (*n* = 8). The asterisk in ***B***, ***C***, and ***I*** indicates a significant difference (*p < 0.05, **p < 0.01, ***p < 0.001). Statistical comparisons were conducted using two-way (***B***, ***C***) or one-way (***I***) repeated measures ANOVA followed by Bonferroni's *post hoc* test. Error bars represent ± SEM. Stim, stimulation; BSR, burst-suppression ratio.

The behavioral emergence from general anesthesia always goes along with the activation of the cortex. To explore the role of BF GABAergic neurons in the regulation of cortical activity, we tested the effect of photostimulating these neurons on cortical EEG under 0.8% and 1.4% isoflurane anesthesia. Under 0.8% isoflurane, which induces steady-state general anesthesia (Yin et al., 2019), the cortical EEG of mice showed obvious slow-wave activity, marked by high amplitude and low frequency, under 0.8% isoflurane anesthesia (Fig. 4*A*). Photostimulation at 20 Hz obviously decreased EEG amplitude and increased EEG frequency, which induced a rapid transformation of cortical EEG from slow-wave activity to a state with low amplitude and high frequency (Fig. 4*A*; Movie 1). EEG spectral analysis showed that brief photostimulation caused an obvious decline in δ power (*F*_(6,56)_ = 91.52, *p* < 0.0001, two-way repeated measures ANOVA followed by Bonferroni's *post hoc* test; 44.13 ± 1.07% vs 21.62 ± 1.84%, *p* < 0.0001), a decline in θ power (25.03 ± 1.40% vs 15.69 ± 1.60%, *p* = 0.0014), an increase in alpha power (16.27 ± 0.66% vs 31.66% ± 1.51, *p* < 0.0001), and an increase in β power (7.05 ± 0.45% vs 22.97 ± 1.71%, *p* = 0.0001; Fig. 4*B*). In mCherry mice, photostimulation did not produce a significant change in EEG δ, θ, α, or β power (Fig. 4*C*). To further illustrate the effect of BF GABAergic activation on EEG power, corresponding power densities were calculated (Fig. 4*D–G*). It is worth noting that 20-Hz photostimulation potently increased 20-Hz cortical activity in the ChR2 group, but not in the mCherry group. The increase in 20-Hz cortical activity was possible because of the direct disinhibition of the cerebral cortex by BF GABAergic neurons (Henny and Jones, 2008; Kim et al., 2015).

Movie 1. In a ChR2-expressing mouse, photostimulation of BF GABAergic neurons with blue light (10 ms, 5 mV, 20 Hz) obviously decreased EEG amplitude and increased EEG frequency, and induced a rapid transformation of cortical EEG from slow-wave activity to a state with low amplitude and high frequency, under 0.8% isoflurane anesthesia. After photostimulation for 120 s, the power percentage of these waves gradually returned to the baseline before the photostimulation (Fig. 4*B*). In mCherry mice, photostimulation did not induce EEG change under isoflurane anesthesia (Movie 2). These results indicate that activation of BF GABAergic neurons promotes cortical activation during steady-state isoflurane anesthesia. 10.1523/JNEUROSCI.0628-22.2023.video.1

Movie 2. In a mCherry-control mouse, photostimulation of BF GABAergic neurons with blue light (10 ms, 5 mV, 20 Hz) did not induce EEG change under 0.8% isoflurane anesthesia. 10.1523/JNEUROSCI.0628-22.2023.video.2

Next, we tested the effect of photostimulating BF GABAergic neurons on cortical activity under 1.4% isoflurane anesthesia, which induces stable burst-suppression oscillation in cortical EEG. The BSR is widely used in animal experiments as one important indicator for assess the depth of anesthesia. Our results showed that, under 1.4% isoflurane, a stable EEG burst suppression mode was observed, with the BSR reaching 81%, indicating that the mice lapsed into a deeper state of general anesthesia. Photostimulation of BF GABAergic neurons rapidly interrupted the burst suppression oscillation of EEG and induced the transformation of cortical EEG from burst suppression oscillation to a low-voltage fast activity mode (Fig. 4*H*; Movie 3). Statistics showed that photostimulation at 20 Hz significantly decreased the BSR (*F*_(1.624,11.37)_ = 44.66, *p* < 0.0001, one-way repeated measures ANOVA followed by Bonferroni's *post hoc* test; pre-stim–stim comparison, 81.41 ± 5.28% vs 25.31 ± 3.85%, *p* = 0.0003; Fig. 4*I*). After 120 s photostimulation, the BSR gradually recovered. In mCherry mice, photostimulation did not induce a BSR change under isoflurane anesthesia (Movie 4). Collectively, the changes in the EEG spectrum and the decrease in BSR indicate that activation of GABAergic BF promotes the activity of the cerebral cortex and attenuates the depth of isoflurane anesthesia.

Movie 3. In a ChR2-expressing mouse, photostimulation of BF GABAergic neurons with blue light (10 ms, 5 mV, 20 Hz) rapidly interrupted the burst-suppression oscillation of EEG, and induced transformation of cortical EEG from burst-suppression oscillation to a low-voltage fast activity mode, under 1.4% isoflurane anesthesia. 10.1523/JNEUROSCI.0628-22.2023.video.3

Movie 4. In a mCherry-control mouse, photostimulation of BF GABAergic neurons with blue light (10 ms, 5 mV, 20 Hz) did not induce BSR change under 1.4% isoflurane anesthesia. 10.1523/JNEUROSCI.0628-22.2023.video.4

### Optogenetic activation of the GABAergic BF-TRN pathway recapitulated behavioral emergence from isoflurane anesthesia

**Figure 5.**
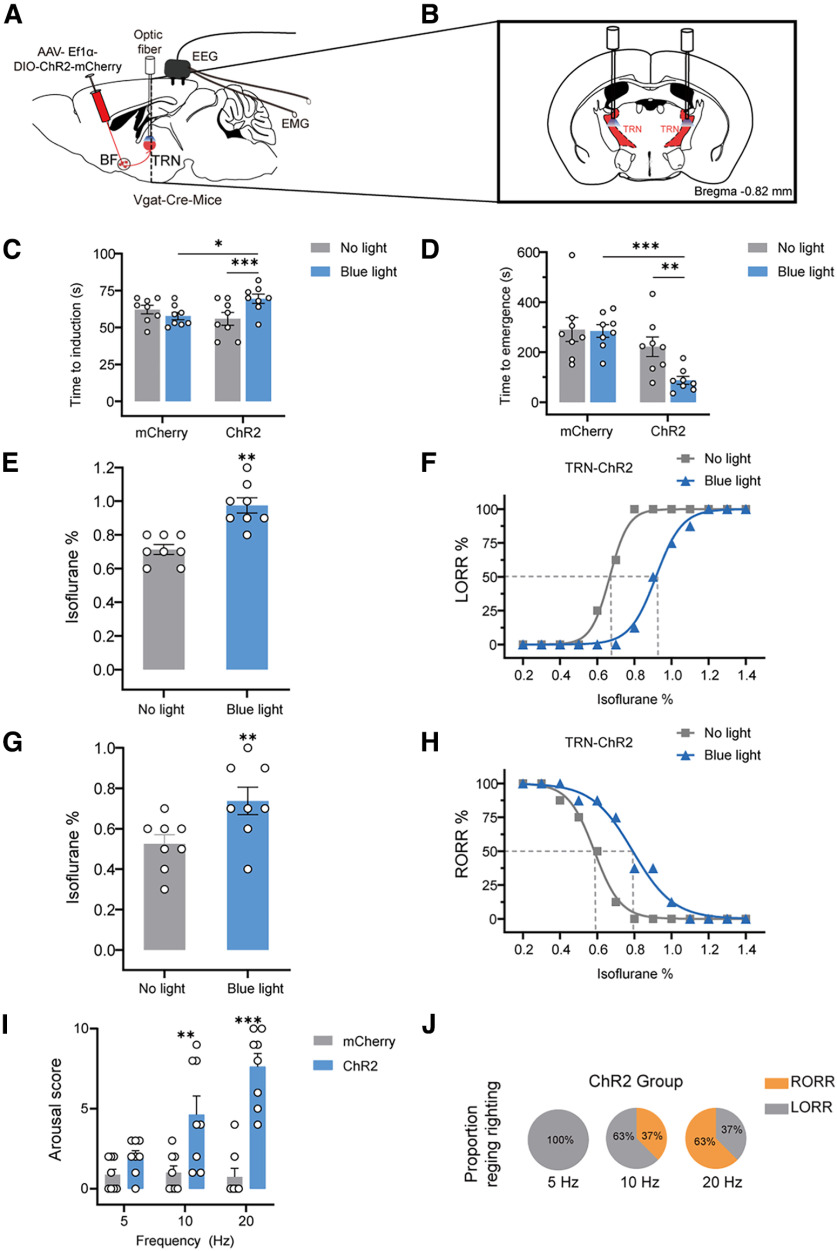
Optogenetic activation of GABAergic BF-TRN pathway delays the induction and facilitates the emergence from isoflurane anesthesia. ***A***, Schematic representation of optogenetic activation of the GABAergic BF-TRN pathway. ***B***, Schematic of coronal section illustrating the location of fiber optic implantation in the TRN following viral microinjection in the BF. ***C***, ***D***, The effect of optogenetic regulation of BF-TRN pathway on LORR and RORR time under 1.4% isoflurane anesthesia (*n* = 8 ChR2 vs *n* = 8 mCherry). ***E***, Photostimulation of BF-TRN pathway increased the isoflurane concentration inducing LORR in ChR2 mice (*n* = 8). ***F***, The dose-response curve shows the percentage of mice showing LORR in response to incremental isoflurane concentration in ChR2 group with or without blue light stimulation (*n* = 8). ***G***, Photostimulation of BF-TRN pathway increased the isoflurane concentration at which ChR2 mice showed RORR (*n* = 8). ***H***, The dose-response curve shows the percentage of mice showing RORR with gradually decreased isoflurane concentration in ChR2 group with or without blue light stimulation (*n* = 8). ***I***, Arousal scores based on the mice behavioral response to BF-TRN pathway photostimulation (5–20 Hz, 5 ms, 60 s) under isoflurane anesthesia (*n* = 8 ChR2 mice or *n* = 8 mCherry mice per group). ***J***, Pie chart shows the proportion ChR2 mice recovering righting reflex after photostimulation of BF-TRN pathway at 5–20 Hz for 60 s (*n* = 8 ChR2 mice per group). The asterisk in ***C–I*** indicates a significant difference (*p < 0.05, **p < 0.01, ***p < 0.001). Statistical comparisons were conducted using Wilcoxon signed-rank test (***E***, ***G***) or two-way repeated measures ANOVA followed by Bonferroni's *post hoc* test (***C***, ***D***, ***I***). Error bars represent mean ± SEM. LORR, loss of righting reflex; RORR, recovery of righting reflex. Additional information to support this figure can be found in the Extended Data Figures 5-1 and 5-2.

The thalamic reticular nucleus (TRN) is a critical nucleus regulating the activity of the thalamus and cortex and has been involved in the regulation of anesthesia (Herrera et al., 2016; Zhang et al., 2019). Neurotomical results illustrated that the TRN receives adequate GABAergic projections from the BF (Agostinelli et al., 2019). We speculated that the TRN may mediate the anti-anesthetic effect of BF GABAergic neurons. To test this hypothesis, we assessed the effect of photostimulating the GABAergic BF-TRN pathway on isoflurane anesthesia, using the same experimental paradigm of Figure 3 AAV-EF1α-DIO-ChR2-mCherry or AAV-EF1α-DIO-mCherry was injected into the BF of Vgat-Cre mice (ChR2 mice or mCherry mice), and the optical fiber was implanted in the TRN (Fig. 5*A*,*B*). We first observed the effect of activation of GABAergic BF-TRN pathway on the induction and emergence under 1.4% isoflurane anesthesia. Our results showed that photostimulation significantly increased the induction time from 56 ± 4 to 70 ± 3 s (*F*_(1,14)_ = 24.93, *p* = 0.0002, two-way repeated measures ANOVA followed by Bonferroni's *post hoc* test; ChR2-mice, *p* = 0.0002; Fig. 5*C*) and decreased the emergence time from 222 ± 39 to 87 ± 16 s (*F*_(1,14)_ = 5.40, *p* = 0.0357, two-way repeated measures ANOVA followed by Bonferroni's *post hoc* test; ChR2-mice, *p* = 0.0081; Fig. 5*D*) in ChR2 mice. In the mCherry mice that received AAV-EF1α-DIO-mCherry injection in the BF, photostimulation did not change the induction or emergence time (Fig. 5*C*,*D*).

10.1523/JNEUROSCI.0628-22.2023.f5-1 Extended Data Figure 5-1 Behavioral responses of Vgat-Cre mice during acute optogenetic activation of GABAergic neurons BF-TRN pathway at 20 Hz under Isoflurane anesthesia. Behavioral responses were undertaken during 60 s of acute photostimulation. Spontaneous movements of the head, tail and limbs, as well as righting reflex and walking status were scored for each mouse. The total score for each mouse depends on the sum of all categories. Download Figure 5-1, DOCX file.

10.1523/JNEUROSCI.0628-22.2023.f5-2 Extended Data Figure 5-2 Presynaptic projection of BF GABAergic neurons to anesthesia-related brain structures. ***A***, Schematic diagram showing the injection of AAV-hSyn-DIO-mGFP-T2A-Synaptophysin-mRuby into the BF of Vgat-Cre mice. ***B***, A representative image of viral expression in the BF of Vgat-Cre mice. Scale bar, 200 μm. ***C–K***, Representative images of mRuby signals. MS, medial septal nucleus; TRN, thalamic reticular nucleus; LHb, lateral habenula nucleus; LH, lateral hypothalamus; SuM, supramammillary nucleus; VTA, ventral tegmental area; DRN, dorsal raphe nucleus; SNc, substantia nigra pars compacta; vlPAG, ventrolateral periaqueductal gray; LPAG, lateral periaqueductal gray; LPB, lateral parabrachial; MPB, medial parabrachial. Scale bar, 200 μm. Download Figure 5-2, TIF file.

Then, we detected the effect of activation of the GABAergic BF-TRN pathway on isoflurane sensitivity. Our results showed that the isoflurane concentration inducing LORR increased from 0.713 ± 0.030% to 0.975 ± 0.045% by 20-Hz photostimulation (*W* = 36, *p* = 0.0078; Fig. 5*E*). Photostimulation significantly increased the isoflurane EC_50_ of LORR from 0.667% (95% confidence interval, 0.654–0.677%) to 0.916% (95% confidence interval, 0.895–0.931%; Fig. 5*F*). Optogenetic activation of the GABAergic BF-TRN pathway also influenced RORR. The isoflurane concentration inducing RORR increased significantly from 0.525 ± 0.045% to 0.738 ± 0.068% (*W* = 36; *p* = 0.0078; Fig. 5*G*). The isoflurane EC_50_ of RORR increased from 0.584% (95% confidence interval, 0.569–0.608%) to 0.791% (95% confidence interval, 0.761–0.833%; Fig. 5*H*).

Next, we investigated the effect of activating the GABAergic BF-TRN pathway on the initiation of behavioral emergence from isoflurane anesthesia. Our results showed that photostimulation strongly promoted mice behavioral responses and significantly increased the arousal scores (Fig. 5*I*). Photostimulation at 20 Hz induced obvious behavioral responses, including body movement (limbs, heads, tails; 8/8), regaining the righting reflex (5/8), and walking (4/8), in ChR2 mice (Extended Data Fig. 5-1). Photostimulation increased the proportion of mice regaining the righting reflex in a frequency-dependent manner (Fig. 5*J*). Photostimulation at 20 Hz induced the regaining of the righting reflex in 63% of mice (Fig. 5*J*). In mCherry mice, photostimulation did not induce obvious behavioral responses in mice under isoflurane anesthesia (Fig. 5*I*). These results clearly illustrate that activation of the GABAergic BF-TRN pathway is sufficient to facilitate behavioral emergence from isoflurane anesthesia.

### Optogenetic activation of the GABAergic BF-TRN pathway recapitulated cortical activation during isoflurane anesthesia

**Figure 6.**
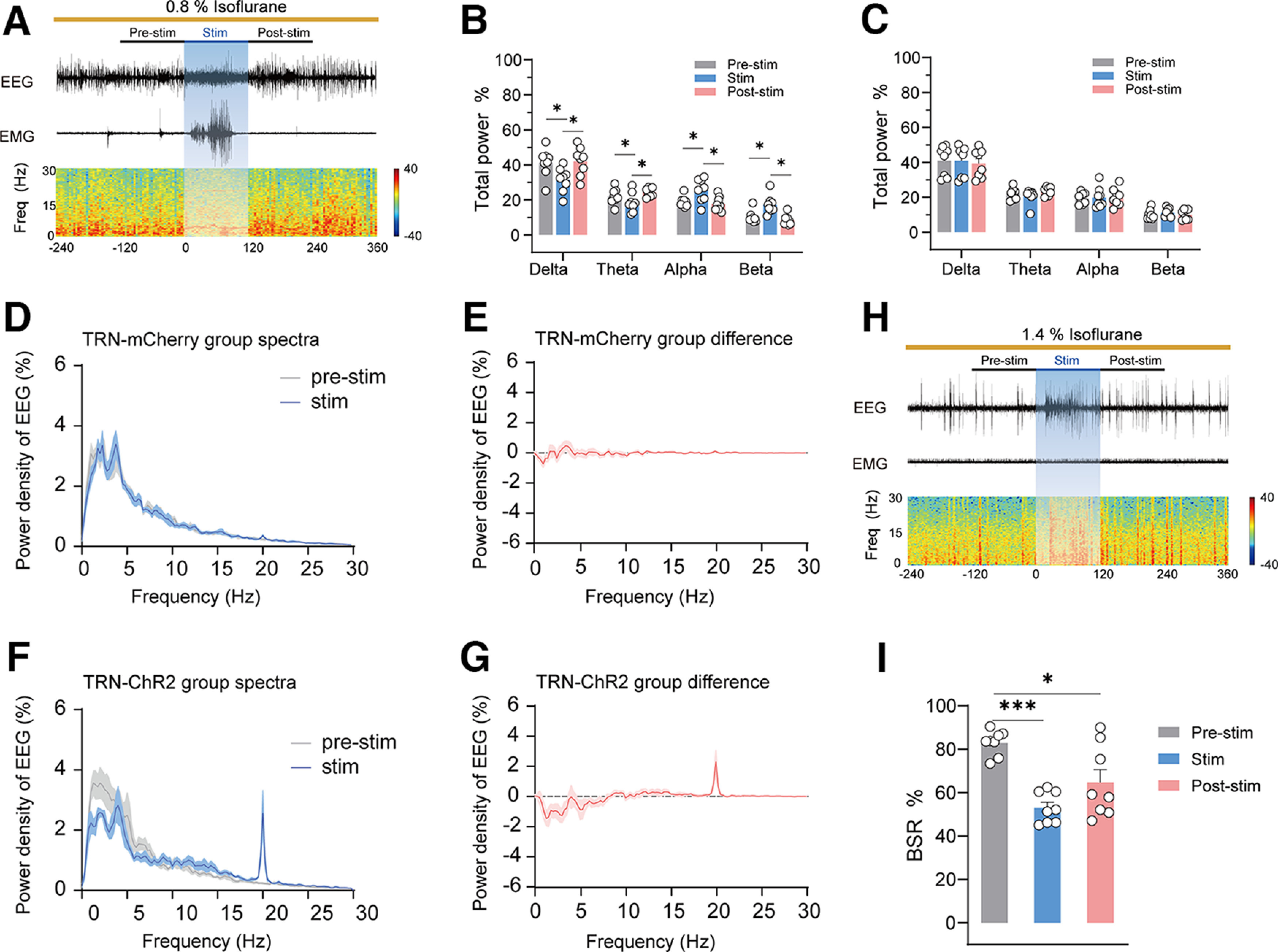
Optogenetic stimulation of GABAergic BF-TRN pathway induces rapid cortical activation during 0.8% and 1.4% isoflurane anesthesia. ***A***, Representative images showing EEG, EMG, and EEG spectrograms of a ChR2 mouse receiving acute photostimulation (20 Hz, 10 ms, 120 s) during 0.8% isoflurane anesthesia. ***B***, Relative EEG power before (gray), during (blue) and after (pink) photostimulation at 20 Hz in TRN-ChR2 mice during isoflurane anesthesia (*n* = 8). ***C***, Relative EEG power before (gray), during (blue), and after (pink) photostimulation at 20 Hz in TRN-mCherry mice during isoflurane anesthesia (*n* = 8). ***D***, Acute photostimulation at 20 Hz does not change normalized power densities of EEG signals in TRN-mCherry mice (*n* = 8). ***E***, Differences of normalized power densities of EEG signals before and during acute photostimulation at 20 Hz in TRN-mCherry mice. ***F***, Acute photostimulation at 20 Hz changes normalized power densities of EEG signals in TRN- ChR2 mice (*n* = 8). ***G***, Differences of normalized power densities of EEG signals before and during acute photostimulation at 20 Hz in TRN-ChR2 mice. ***H***, Representative images showing EEG, EMG, and EEG spectrograms of a ChR2 mouse receiving acute photostimulation (20 Hz, 10 ms, 120 s) during 1.4% isoflurane anesthesia. ***I***, Relative BSR before (gray), during (blue), and after (pink) photostimulation at 20 Hz in ChR2 mice during 1.4% isoflurane anesthesia (*n* = 8). The asterisk in ***B***, ***C***, and ***I*** indicates a significant difference (*p < 0.05, ***p < 0.001). Statistical comparisons were conducted using two-way (***B***, ***C***) or one-way (***I***) repeated measures ANOVA followed by Bonferroni's *post hoc* test. Error bars represent ± SEM. Stim, stimulation; BSR, burst-suppression ratio.

After verifying the behavioral response induced by the activation of the GABAergic BF-TRN pathway, we further investigated its effect on cortical activity using the same experimental paradigm of Figure 4. Similar to the results of BF cell body activation, 20-Hz photostimulation of TRN terminals rapidly interrupted slow-wave activity and induced high-frequency and low-amplitude EEG activity under 0.8% isoflurane anesthesia (Fig. 6*A*). EEG spectral analysis showed that brief photostimulation induced a notable decline in δ power (*F*_(6,56)_ = 15.93, *p* < 0.0001, two-way repeated measures ANOVA followed by Bonferroni's *post hoc* test; 41.36 ± 2.85% vs 31.46 ± 2.47%, *p* = 0.0141), a decline in θ power (22.13 ± 1.58% vs 18.57 ± 1.96%, *p* = 0.0403), an increase in alpha power (19.05 ± 1.05% vs 25.45 ± 2.34%, *p* = 0.0468), and an increase in β power (10.25 ± 1.22% vs 17.24 ± 1.84%, *p* = 0.0180; Fig. 6*B*). In mCherry mice, photostimulation did not produce a significant change in EEG δ, θ, α, or β power (Fig. 6*C*). To further illustrate the effect of activating the BF-TRN pathway on EEG power, corresponding power densities were calculated (Fig. 6*D–G*). After the cessation of photostimulation, the power percentage of these waves gradually returned to the baseline before the photostimulation (pre-stim–post-stim comparison *p* > 0.05; Fig. 6*B*). These results indicate that activation of the GABAergic BF-TRN pathway promotes cortical activation during steady-state isoflurane anesthesia.

Next, we tested the effect of photostimulating GABAergic BF-TRN pathway on cortical activity under 1.4% isoflurane anesthesia. Photostimulation of TRN terminals rapidly interrupted the burst suppression oscillation of EEG and induced low-voltage fast activity of EEG (Fig. 6*H*). Statistics showed that 20-Hz photostimulation significantly decreased the BSR (*F*_(2,14)_ = 19.58, *p* < 0.0001, one-way repeated measures ANOVA followed by Bonferroni's *post hoc* test; pre-stim–stim comparison, 82.95 ± 2.02% vs 53.02 ± 2.53%, *p* < 0.0001; Fig. 6*I*). The BSR was gradually restored after the cessation of photostimulation. Collectively, these results indicate that activation of the GABAergic BF-TRN pathway is sufficient to facilitate cortical emergence from isoflurane anesthesia.

## Discussion

Previous studies showed that wake-related brain regions are involved in general anesthesia regulation. Transient optogenetic stimulation of nucleus accumbens neurons expressing dopamine D1 receptors causes cortical activation and behavioral emergence from sevoflurane anesthesia (Bao et al., 2021). Activation of parabrachial nucleus (PBN) glutamatergic neurons accelerates the emergence from general anesthesia (T.X. Wang et al., 2019). BF GABAergic neurons have been demonstrated to play a significant role in regulating wakefulness, chemogenetic activation of BF GABAergic neurons induced sustained wakefulness with high-frequency cortical rhythms (Anaclet et al., 2015). In the current study, our results illustrated that BF GABAergic neurons facilitate behavioral and cortical emergence from general anesthesia. This evidence supports the hypothesis that general anesthetics and sleep–wake behaviors may share similar neural pathways.

Prior research demonstrated that the cholinergic neurons in the BF play an important role in controlling cortical activity and arousal (Anaclet et al., 2015; M. Xu et al., 2015) and facilitate wakefulness by acting on adjacent noncholinergic neurons, especially cortically projecting GABAergic neurons (Zant et al., 2016). *In vivo* studies showed that optogenetic activation of cholinergic neurons increased wakefulness in mice and increased acetylcholine levels locally in the BF, and the enhanced wakefulness induced by BF cholinergic activation was abolished by simultaneous reverse microdialysis of cholinergic receptor antagonists into the BF (Zant et al., 2016). *In vitro* studies showed that BF cholinergic neurons strongly excited neighboring GABAergic neurons, especially cortically projecting GABAergic neurons (C. Yang et al., 2014). Optogenetic activation of BF cholinergic neurons notably excited neighboring cortically projecting GABAergic neurons primarily through nicotinic acetylcholine receptors (M. Xu et al., 2015). A recent study revealed that BF cholinergic neurons also participate in the regulation of general anesthesia (L. Wang et al., 2021). Optogenetic activation of BF cholinergic neurons accelerated emergence from propofol-induced unconsciousness, decreased δ power, and increased β and γ power in the medial prefrontal cortex (L. Wang et al., 2021). Considering the similarity of the sleep–wake pathway and anesthesia pathway, we speculate that cholinergic BF may regulate anesthesia emergence, at least partially, through the excitation of local GABAergic neurons, especially cortically projecting GABAergic neurons, similar to the mechanism through which cholinergic BF neurons regulate wakefulness.

In a previous study, Shuang Cai and colleagues investigated the roles of two subtypes of BF GABAergic neurons, parvalbumin-positive neurons, and somatostatin-positive neurons, in general anesthesia using genetic lesion and chemogenetic approaches (Cai et al., 2021). Their results showed that BF parvalbumin-positive neurons only play a limited and nuanced role in isoflurane anesthesia, while somatostatin-positive neurons promote the anesthetic effect of isoflurane (Cai et al., 2021). These results are quite different from ours, which showed that activation of BF GABAergic neurons potently promoted behavioral and cortical emergence from isoflurane anesthesia. The contradictory results may be because of the heterogeneity of BF GABAergic neurons (C. Yang et al., 2017). BF GABAergic neurons contain a variety of subtypes, including parvalbumin-positive neurons (McKenna et al., 2013), somatostatin-positive neurons (M. Xu et al., 2015), neurokinin B receptor neurons (Furuta et al., 2004), potassium channel Kv2.2 neurons (Hermanstyne et al., 2010), and neuropeptide Y neurons (Zaborszky et al., 2012). Different subtypes of BF GABAergic neurons may play different roles in general anesthesia. It seems that some subtypes, excluding parvalbumin-positive neurons and somatostatin-positive neurons, may mediate the anti-anesthetic effect of BF GABAergic neurons. The specific role of each subtype of BF GABAergic neurons in general anesthesia needs further research.

The TRN is a thin sheet of GABAergic structures encasing the thalamus. It receives afferent fibers from the cerebral cortex and subcortical regions and mainly sends fibers to the thalamus, which exerts strong inhibition control over thalamocortical relay cells. Previous studies have demonstrated that the TRN is involved in the regulation of the sleep–wake cycle (Lewis et al., 2015; Ni et al., 2016). Optogenetic stimulation of cholinergic inputs to the TRN, which activates TRN GABAergic neurons, promotes sleep and protects NREM sleep in mice (Ni et al., 2016). The results of whole-cortex electrophysiology recording combined with optogenetics showed that local tonic activation of the TRN rapidly induces slow-wave activity in a spatially restricted region of the cortex (Lewis et al., 2015). Recently, the TRN was shown to be involved in the regulation of general anesthesia (Herrera et al., 2016; Zhang et al., 2019). Chemogenetic activation of noradrenergic terminals in the TRN delays emergence from propofol anesthesia in mice (Zhang et al., 2019). Optogenetic activation of the GABAergic projection from the lateral hypothalamus (LH) to the TRN induced cortical arousal and emergence from isoflurane anesthesia (Herrera et al., 2016). *In vivo* electrophysiological experiments have shown that optogenetic stimulation of GABAergic projection from the LH to the TRN causes a temporary but significant decrease in TRN neuron activity, demonstrating direct inhibition of TRN neurons by the GABAergic projection from the LH to the TRN (Herrera et al., 2016). Herrera et al. (2016) suggested that the GABAergic LH-TRN pathway may exert an anti-anesthetic effect through the inhibition of GABAergic TRN neurons, which removes the inhibition of the thalamocortical pathway by the GABAergic TRN and therefore promotes the activation of the cerebral cortex. It is worth noting that the TRN receives GABAergic inputs not only from the LH but also from other brain regions, such as the BF. It is conceivable that the inhibition of GABAergic TRN and consequent disinhibition of the thalamocortical pathway may be the mechanism underlying how the GABAergic BF-TRN pathway facilitates emergence from general anesthesia, similar to the mechanism of the GABAergic LH-TRN pathway controlling consciousness and arousal.

In the current study, our results showed that the anti-anesthetic effect of the GABAergic BF-TRN pathway cannot fully imitate the anti-anesthetic effect of the GABAergic BF. The effect of activating the GABAergic BF-TRN pathway on facilitating behavioral and cortical emergence was obviously weaker than the effect of activating the GABAergic BF. It is reasonable to infer that the GABAergic BF, as a key structure controlling emergence from general anesthesia, may mediate the anti-anesthetic effect via multiple downstream targets in addition to the TRN. To better understand the possible downstream targets of BF GABAergic neurons, we traced the projection of these neurons using AAV-hSyn-DIO-mGFP-T2A-Synaptophysin-mRuby, which makes the cell body of BF GABAergic neurons express membrane-bound GFP (mGFP) and the synaptic terminals of these neurons express synaptophysin-mRuby (W. Xu and Südhof, 2013). Four weeks after the injection of AAV-hSyn-DIO-mGFP-T2A-Synaptophysin-mRuby into the bilateral BF of Vgat-mice (Extended Data Fig. 5-2*A*,*B*), we checked the whole-brain projection pattern of BF GABAergic neurons. In addition to the TRN, the mRuby signals were observed in many anesthesia-related structures, such as the habenula nucleus, lateral hypothalamus, supramammillary nucleus, ventral tegmental area, and periaqueductal gray (Extended Data Fig. 5-2*C–K*). Our projection-tracing results, together with previous neuroanatomical studies (Do et al., 2016; Agostinelli et al., 2019), demonstrate that the GABAergic BF indeed projects to multiple anesthesia-related structures, including the TRN. This one-to-many circuit arrangement may help the GABAergic BF to communicate with multiple downstream targets simultaneously to produce instant arousal from general anesthesia. The specific roles of other downstream targets of the GABAergic BF in general anesthesia need to be further investigated in future studies.
