# Supplementary material for: Facilitation of Behavioral and Cortical Emergence from Isoflurane Anesthesia by GABAergic Neurons in Basal Forebrain
Source: J Neurosci. 2023 Apr 19;43(16):2907–20. doi: 10.1523/JNEUROSCI.0628-22.2023 (PMC10124952; doi:10.1523/JNEUROSCI.0628-22.2023)
Supplement: Extended Data 1 — Supplemental materials and methods. Details of the methods of fiber photometry recording, estimation of induction and emergence, arousal scoring during photostimulation, burst suppression ratio (BSR) analysis, and immunofluorescence. Download Extended Data 1, DOCX file. [file ns-JN-RM-0628-22-s05.docx]

**Fiber photometry recording instruments**

We used a two-color fiber recording system (Inper, China) to record specific types of neural cell activity information in specific brain regions of mice. The outgoing light of the blue light source (470 nm) and purple light source (410nm) was combined into a beam through filtering, quasi value and two-phase color mirror, converged by 20x objective lens and coupled to the same multimode fiber. The resulting GCaMP6m fluorescence after the excitation light was projected into a specific brain region of the mouse passed through a band-pass filter and focused on the sensor of a CMOS camera. The Inper processor imaged the fiber ended. Finally, Inper analysis software was used to calculate the RIO mean on the end surface of the fiber.

The data recorded by the two-color optical fiber recording system (Inper, China) were further analyzed by Matlab R2019b software. The fluorescence signal F was converted to ΔF/F = (F-F_baseline_) / F baseline, where F_baseline_ was either during the 30 min recording period before isoflurane on or during the induction period (- 300 to 0 seconds, baseline before anesthesia), or during the emergence period (-300 to 0 seconds, unconsciousness anesthesia baseline).

**Estimation of induction and emergence in the optogenetic and chemogenetic experiments**

In optogenetic experiments, the mice were connected to the laser stimulator through optical fiber and placed in a chamber filled with 1.4% isoflurane gas while the laser stimulator was turned on to continuously apply light pulses (10ms, 5mV, 20Hz) until the mice developed LORR. The time from exposure to 1.4% isoflurane gas to the development of LORR was recorded. After 30 min of anesthesia, the mice were removed from the chamber and exposed to air, while the light stimulator was turned on until the mice developed RORR, and then the light stimulation was stopped. The time from exposure to air to the onset of RORR was recorded.

In the experiments investigating the effect of photostimulation on the dose-response curve of LORR, isoflurane was initially delivered at a concentration of 0.5%, followed by 120 s of photostimulation after 15 min, and if no LORR occurred, the isoflurane concentration in the chamber was increased 0.1%. Repeat steps after 15 min until mice develop LORR. Similarly, in experiments to determine dose-response curves for RORR, isoflurane was initially delivered at a concentration of 1.4% and tapered by 0.1% every 15 minutes until mice developed RORR during 120 s of photostimulation.

**Arousal scoring during photostimulation**

The spontaneous movements of the head, tail, and limbs were scored as three levels: absent (0), mild (1) and moderate (2) in intensity. Righting was scored as 0 if the mouse remained with LORR, and 2 if the mouse recovered its righting reflex during the 60 s acute photostimulation. Walking after RORR was scored as follows: 0 = no further movements; 1 = crawled without the abdomen off the chamber bottom; and 2 = walked with the abdomen off the chamber bottom. The total score for each mouse depended on the sum of all categories.

**BSR analysis**

EEG signals were processed by using the Hilbert transform to calculate the instantaneous amplitude and a visually-based threshold was set in the transformed signal to segment burst and suppression states for each mouse. A binary series was applied for BSR calculation, with the value being 1 if the transformed signal of EEG was outside the defined threshold and being 0 if it was within the threshold. The minimum duration of burst and suppression periods was set to 0.5 s.

**Immunofluorescence**

Firstly, mice were deeply anaesthetized with [pentobarbital](javascript:;) [sodium](javascript:;). After mice were [unconsciousness](javascript:;), they were intracardially perfused consecutively with cold phosphate-buffered saline (PBS) and 4% paraformaldehyde (PFA). Immediately, brains were moved and post-fixed in the 4% PFA for about 6 hours, and following soaked in 20% and 30% sucrose for cryoprotection. Brain samples were embedded with OCT and stored in -80℃ freezer. The brains were sectioned coronally into 30$\mu$m slices in the cryostat (CM1950, LEICA) after embedding and freezing. The sections were washed three times with 0.01 M PBS to remove the embedding OCT, following be incubated in 0.7% TritonX-100 for 1 hour to permeate cytomembrane. After permeation the slices were firstly incubated with primary antibody in PBST (1:1000, Ab190289, USA) for 24 hours and 488nm-labeled secondary antibody (1:1000, 111-545-003, Jackson) for 2 hours with three times PBS washing during the interval. Images were captured by a fluorescence microscope (DMi8, Leica) using different objectives and then processed by Adobe Illustrate.
