# Supplementary figures and images for: Facilitation of Behavioral and Cortical Emergence from Isoflurane Anesthesia by GABAergic Neurons in Basal Forebrain
Source: J Neurosci. 2023 Apr 19;43(16):2907–20. doi: 10.1523/JNEUROSCI.0628-22.2023 (PMC10124952; doi:10.1523/JNEUROSCI.0628-22.2023)

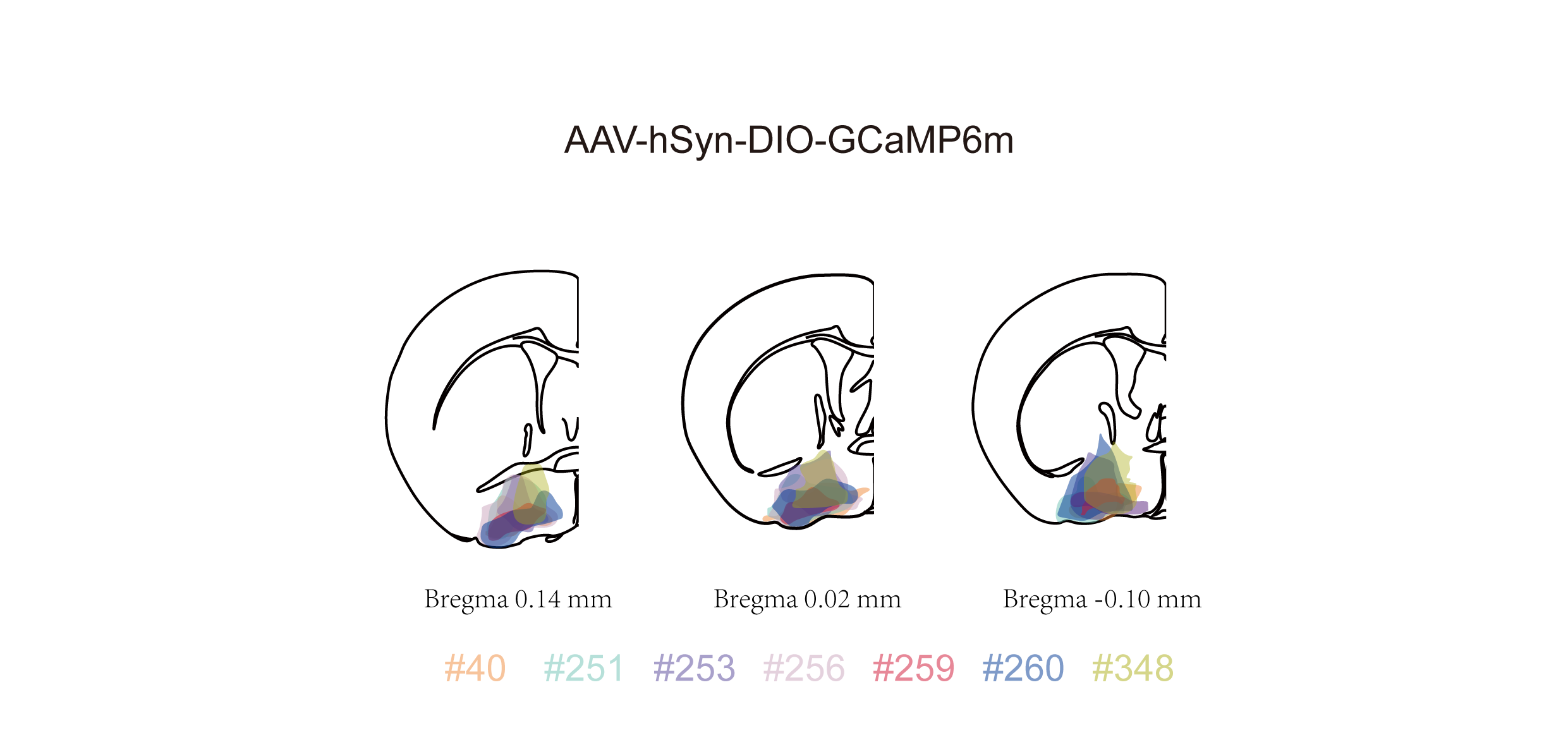

Supplement: Extended Data Figure 1-1 — Drawings of superimposed expression of AAV-hSyn-DIO-GCaMP6m in the BF. AAV-hSyn-DIO-GCaMP6m was injected into the BF of Vgat-Cre mice, and expression of GCaMP6m was checked after behavioral testing (n = 7, indicated with different colors). Download Figure 1-1, TIF file. [file ns-JN-RM-0628-22-s06.tif]

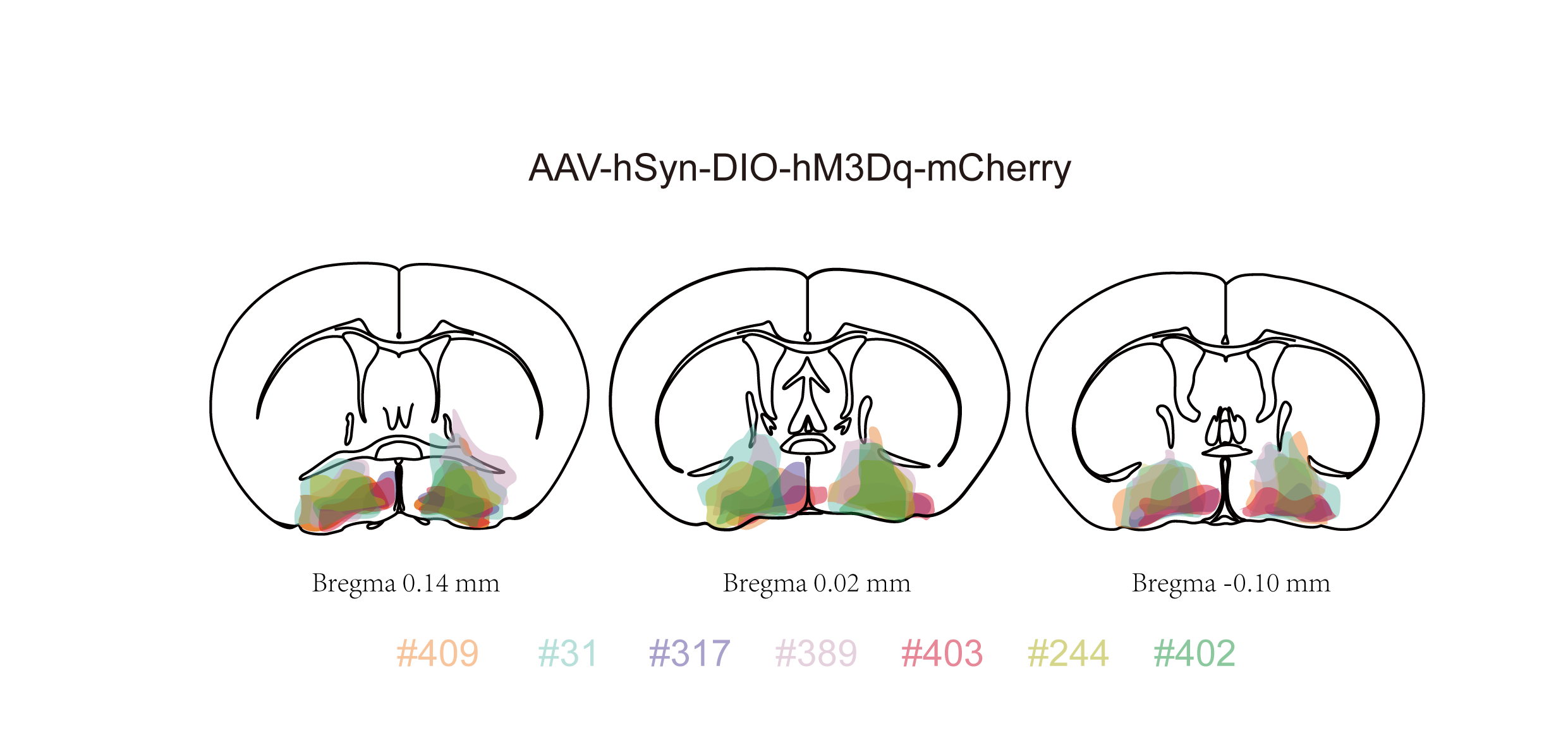

Supplement: Extended Data Figure 2-1 — Drawings of superimposed expression of AAV-hSyn-DIO-hM3Dq-mCherry in the BF. AAV-hSyn-DIO-hM3Dq-mCherry was injected into the BF of Vgat-Cre mice, and the expression of hM3Dq was checked after behavioral testing (n = 7, indicated with different colors). Download Figure 2-1, TIF file. [file ns-JN-RM-0628-22-s07.tif]

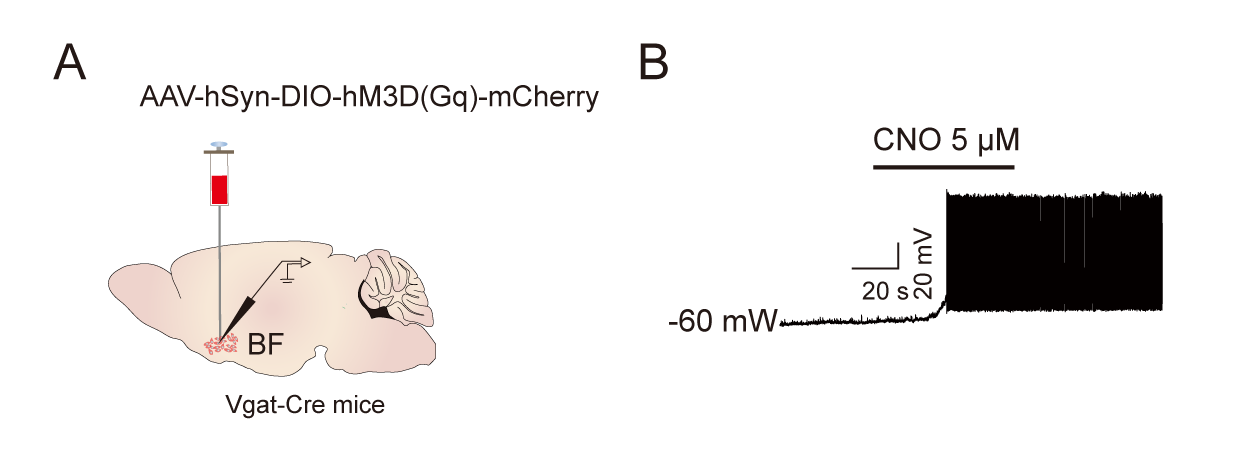

Supplement: Extended Data Figure 2-2 — In vitro electrophysiological results confirm the activation of BF GABAergic neurons by chemogenetic approaches. A, Schematic diagram of injection of AAV-hSyn-DIO-hM3DGq-mCherry into the BF of Vgat-Cre mice to prepare brain slices in vitro electrophysiological experiments. B, Representative voltage tracer shows that bath application of CNO increases firing rate in hM3Dq-expressing BF GABAergic neurons. Download Figure 2-2, TIF file. [file ns-JN-RM-0628-22-s08.tif]

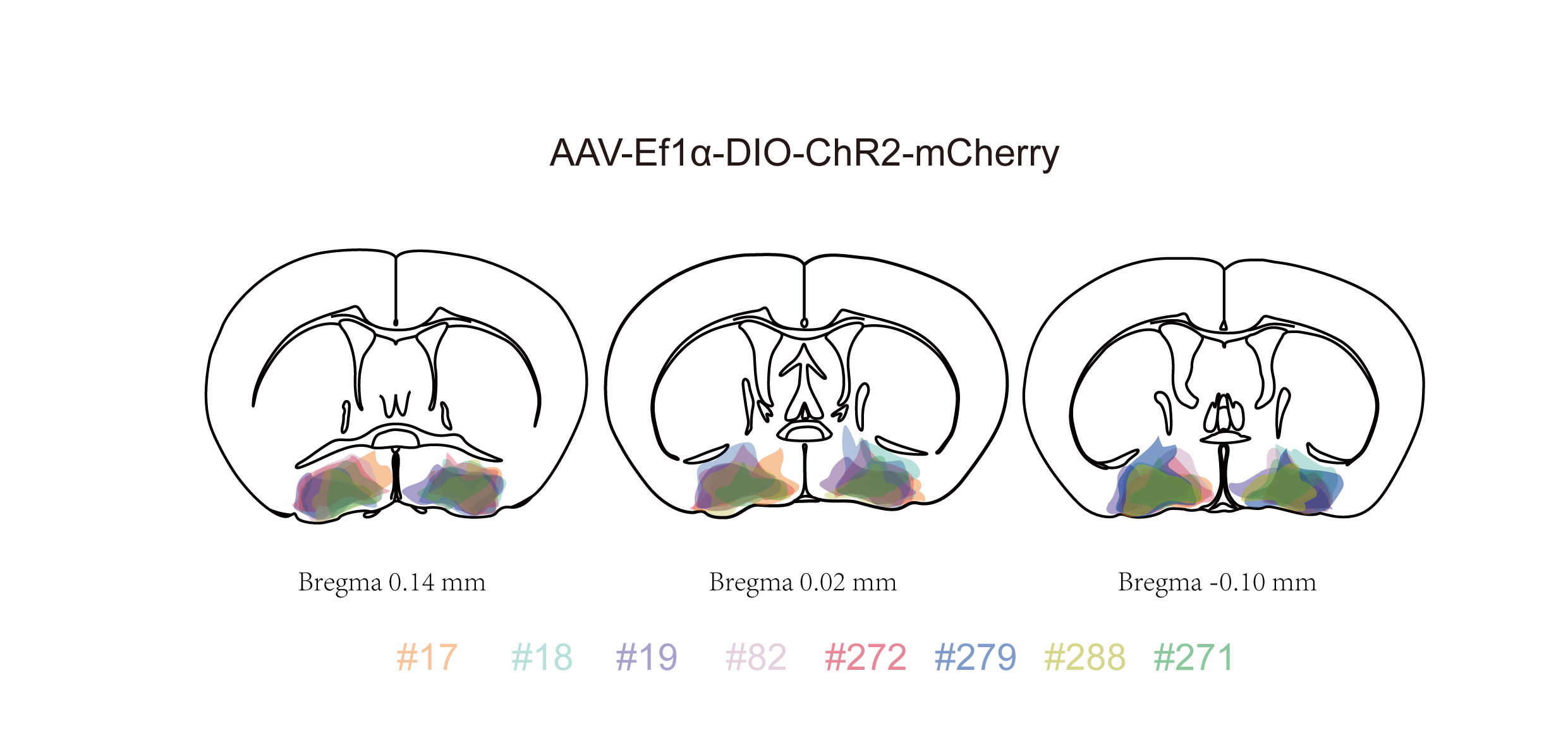

Supplement: Extended Data Figure 3-1 — Drawings of superimposed expression of AAV-Ef1α-DIO-ChR2-mCherry in the BF. AAV-Ef1α-DIO-ChR2-mCherry was injected into the BF of Vgat-Cre mice, and the expression of ChR2 was checked after behavioral testing (n = 8, indicated with different colors). Download Figure 3-1, TIF file. [file ns-JN-RM-0628-22-s09.tif]

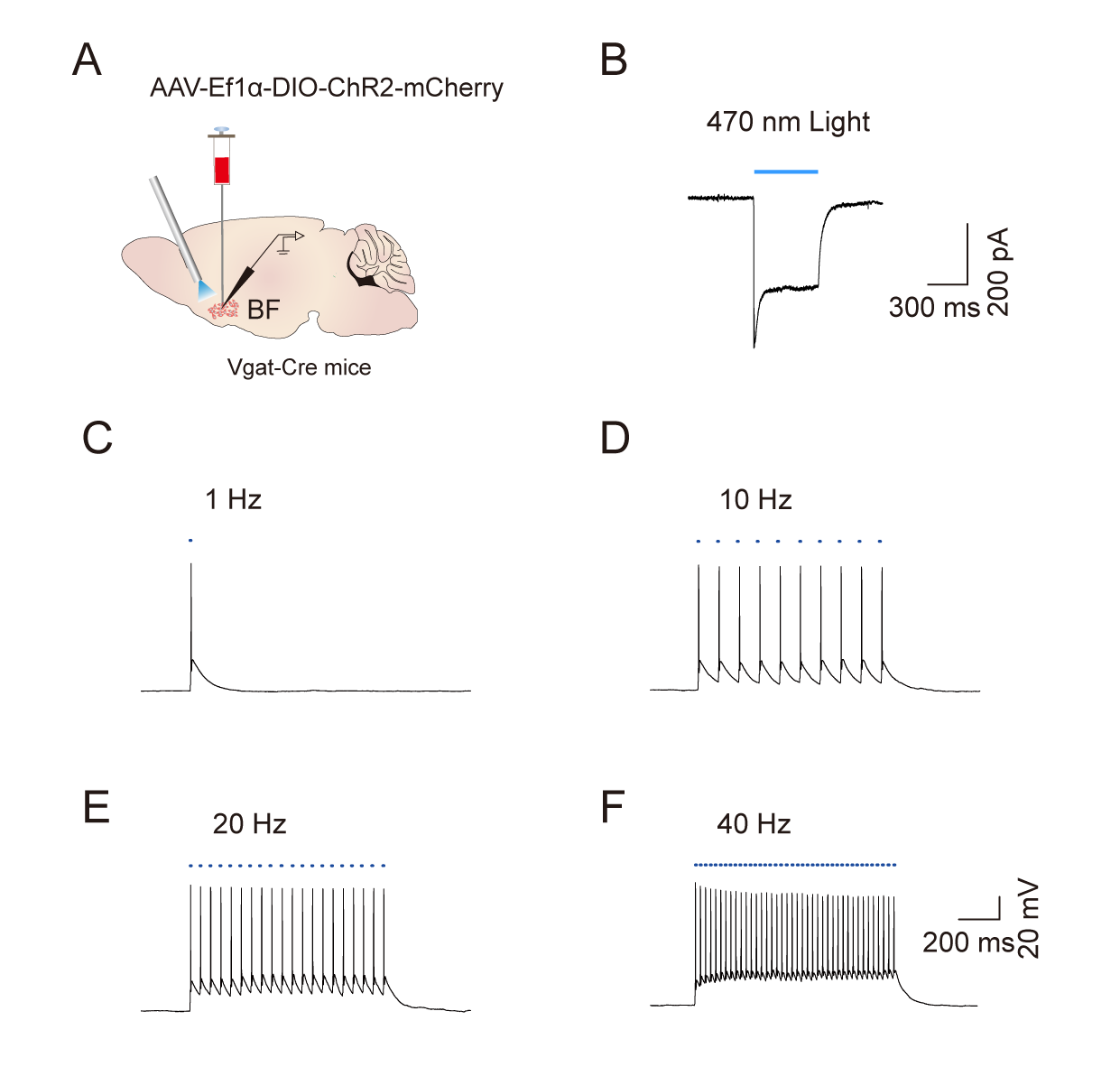

Supplement: Extended Data Figure 3-2 — In vitro electrophysiological results confirm the activation of BF GABAergic neurons by optogenetic approaches. A, Schematic diagram of injection of AAV-EF1α-DIO-ChR2-mCherry into the BF of Vgat-Cre mice to prepare brain slices in vitro electrophysiological experiments. B, Representative current tracer shows that 473-nm blue light stimulation induces a depolarization current in ChR2-expressing BF GABAergic neurons. C–F, Representative current-clamp recording results show that neuronal firing of ChR2-expressing BF GABAergic neurons was reliably evoked by optogenetic stimulation at different frequencies. Download Figure 3-2, TIF file. [file ns-JN-RM-0628-22-s10.tif]

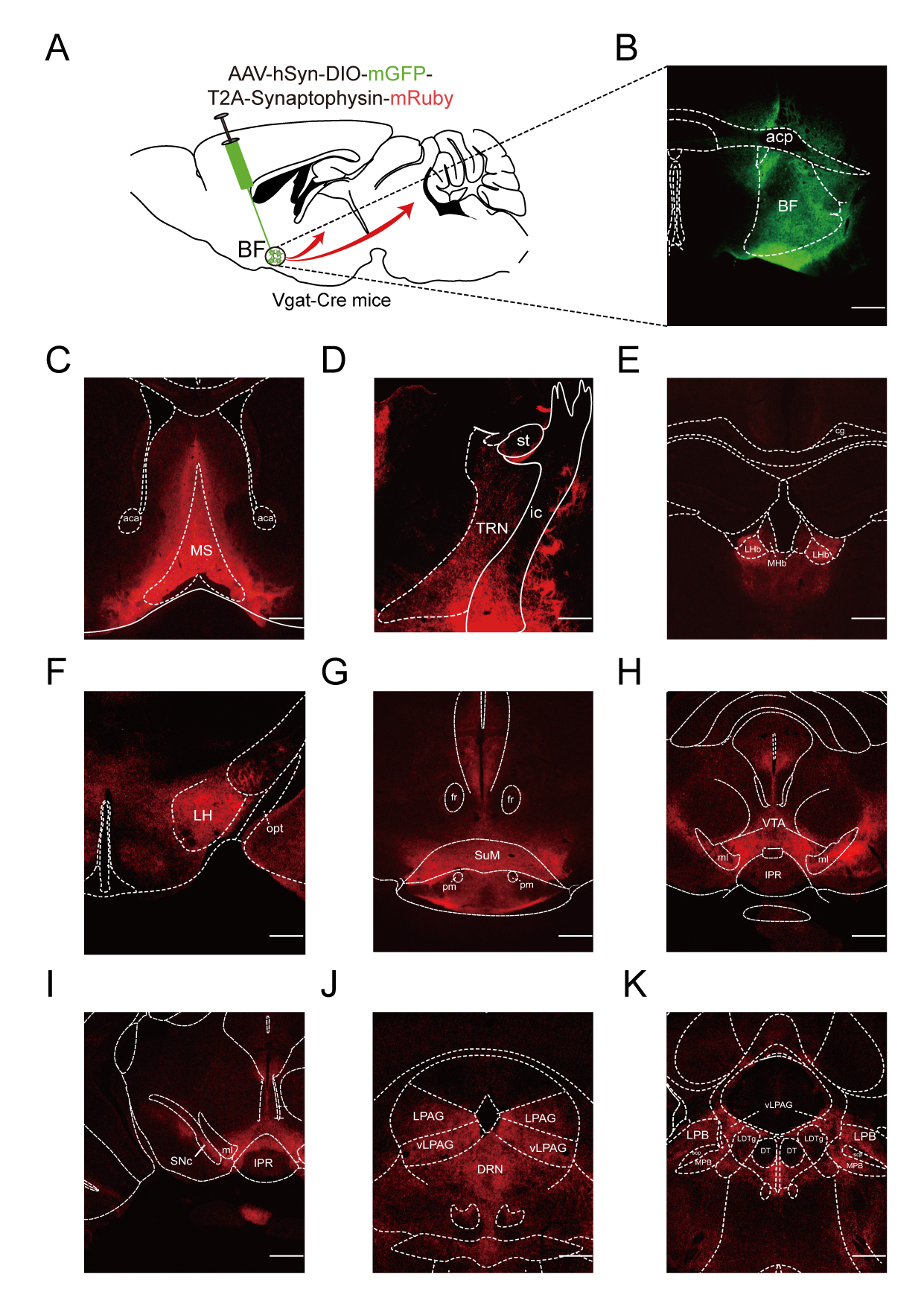

Supplement: Extended Data Figure 5-2 — Presynaptic projection of BF GABAergic neurons to anesthesia-related brain structures. A, Schematic diagram showing the injection of AAV-hSyn-DIO-mGFP-T2A-Synaptophysin-mRuby into the BF of Vgat-Cre mice. B, A representative image of viral expression in the BF of Vgat-Cre mice. Scale bar, 200 μm. C–K, Representative images of mRuby signals. MS, medial septal nucleus; TRN, thalamic reticular nucleus; LHb, lateral habenula nucleus; LH, lateral hypothalamus; SuM, supramammillary nucleus; VTA, ventral tegmental area; DRN, dorsal raphe nucleus; SNc, substantia nigra pars compacta; vlPAG, ventrolateral periaqueductal gray; LPAG, lateral periaqueductal gray; LPB, lateral parabrachial; MPB, medial parabrachial. Scale bar, 200 μm. Download Figure 5-2, TIF file. [file ns-JN-RM-0628-22-s13.tif]
