# Supplementary material for: Facilitation of Behavioral and Cortical Emergence from Isoflurane Anesthesia by GABAergic Neurons in Basal Forebrain
Source: J Neurosci. 2023 Apr 19;43(16):2907–20. doi: 10.1523/JNEUROSCI.0628-22.2023 (PMC10124952; doi:10.1523/JNEUROSCI.0628-22.2023)
Supplement: Extended Data Figure 5-1 — Behavioral responses of Vgat-Cre mice during acute optogenetic activation of GABAergic neurons BF-TRN pathway at 20 Hz under Isoflurane anesthesia. Behavioral responses were undertaken during 60 s of acute photostimulation. Spontaneous movements of the head, tail and limbs, as well as righting reflex and walking status were scored for each mouse. The total score for each mouse depends on the sum of all categories. Download Figure 5-1, DOCX file. [file ns-JN-RM-0628-22-s12.docx]

| **Group** | **Leg movement** | **Head movement** | **Tail movement** | **Righting** | **Walking** | **Total score** |
| --- | --- | --- | --- | --- | --- | --- |
| 1-ChR2 | 2 | 2 | 2 | 2 | 0 | 8 |
| 2-ChR2 | 2 | 2 | 2 | 2 | 1 | 9 |
| 3-ChR2 | 2 | 2 | 2 | 0 | 0 | 6 |
| 4-ChR2 | 2 | 2 | 2 | 2 | 1 | 9 |
| 5-ChR2 | 1 | 2 | 2 | 0 | 0 | 5 |
| 6-ChR2 | 2 | 2 | 0 | 0 | 0 | 4 |
| 7-ChR2 | 2 | 2 | 2 | 2 | 2 | 10 |
| 8-ChR2 | 2 | 2 | 2 | 2 | 2 | 10 |
| 1-mCherry | 0 | 0 | 0 | 0 | 0 | 0 |
| 2-mCherry | 0 | 0 | 0 | 0 | 0 | 0 |
| 3-mCherry | 0 | 0 | 0 | 0 | 0 | 0 |
| 4-mCherry | 0 | 2 | 0 | 0 | 0 | 2 |
| 5-mCherry | 0 | 2 | 2 | 0 | 0 | 4 |
| 6-mCherry | 0 | 0 | 0 | 0 | 0 | 0 |
| 7-mCherry | 0 | 0 | 0 | 0 | 0 | 0 |
| 8-mCherry | 0 | 0 | 0 | 0 | 0 | 0 |
